# Time in Range and Adverse Outcomes in Type 2 Diabetes: A Quantitative Synthesis

**DOI:** 10.3390/jcm15145713

**Published:** 2026-07-21

**Authors:** Furong Qu, Qinbo Yang, Qingyue Zeng, Zhipeng Li, Jing Li

**Affiliations:** 1 General Practice Ward/International Medical Center Ward, General Practice Medical Center, West China Hospital, Sichuan University, Chengdu 610000, China; qufurong66@wchscu.cn (F.Q.);; 2 Department of Nephrology, West China Hospital, Sichuan University, Chengdu 610000, China; 3 Department of Endocrinology and Metabolism, West China Hospital, Sichuan University, Chengdu 610000, China

**Keywords:** type 2 diabetes mellitus, time in range, continuous glucose monitoring, systematic review, diabetes complications

## Abstract

**Objective:** We aimed to quantify the prognostic value of glucose monitoring-derived time in range (TIR), including continuous glucose monitoring (CGM), flash glucose monitoring (FGM), and fingertip capillary glucose monitoring (FCGM), for predicting adverse clinical outcomes in patients with type 2 diabetes mellitus (T2DM). **Research Design and Methods:** PubMed, Embase, and the Cochrane Central Register of Controlled Trials (CENTRAL, via OVID) were systematically searched from 2017 to November 2025 for studies evaluating the risk of all clinically relevant outcomes associated with different TIRs in T2DM. Extracted data were standardized to evaluate the effect of a 10% increment in TIR. Pooled estimates were calculated using inverse-variance random-effects models incorporating dose–response analysis. The certainty of evidence was evaluated using the Grading of Recommendations, Assessment, Development, and Evaluations (GRADE) framework. **Results:** Twenty-four observational studies involving 20 distinct associations and 35,916 participants were included. Dose–response meta-analyses were conducted for nine associations. The results showed that each 10% increment in TIR was significantly associated with a reduced risk of multiple adverse complications, including all-cause mortality (odd ratio [OR] = 0.88, 95% confidence interval [CI]: 0.82–0.93), vision-threatening diabetic retinopathy (OR = 0.93, 95% CI: 0.87–0.98), diabetic retinopathy (OR = 0.92, 95% CI: 0.89–0.95), lower extremity atherosclerotic disease (OR = 0.86, 95% CI: 0.82–0.91), diabetic peripheral neuropathy (OR = 0.77, 95% CI: 0.71–0.84), and cardiovascular autonomic neuropathy (OR = 0.81, 95% CI: 0.68–0.97). In contrast, the associations for albuminuria (KDIGO [Kidney Disease: Improving Global Outcomes] A2 and A3) and amputation did not reach statistical significance in the primary meta-analysis. **Conclusions:** In conclusion, each 10% increment in TIR is consistently associated with a reduced risk of mortality and various micro- and macrovascular complications in T2DM. These findings suggest TIR as a robust prognostic indicator and actionable therapeutic target in diabetes management.

## 1. Introduction

The contemporary paradigm of diabetes management has shifted toward an outcome-oriented strategy that prioritizes the reduction in cardiovascular and kidney complications to improve long-term prognosis [1,2]. Despite this shift, metabolic stability remains the cornerstone of evidence-based care in the management of diabetes. Persistent hyperglycemia is a well-established driver of acute crises, such as diabetic ketoacidosis and hyperosmolar hyperglycemic state, as well as the progressive development of microvascular and macrovascular complications [2]. Glycated hemoglobin (HbA1c) has long been regarded as the gold-standard surrogate marker for predicting outcomes in type 2 diabetes [3,4,5].

However, as a measure of average glycemic exposure over approximately three months, HbA1c fails to capture short-term glycemic fluctuations or glucose variability. These transient excursions may independently contribute to adverse clinical outcomes, explaining why HbA1c alone often fails to account for inter-individual differences in complication risks [6,7,8]. The widespread implementation of continuous glucose monitoring (CGM) and flash glucose monitoring (FGM) has introduced more granular metrics into clinical practice [9,10]. Among these, time in range (TIR), defined for non-pregnant adults as the duration or percentage of time spent within 70 to 180 mg/dL (3.9 to 10.0 mmol/L), has emerged as a clinically intuitive and practical indicator [11]. Several cohort studies have suggested that TIR may be more strongly associated with certain adverse outcomes than HbA1c [12,13,14]. Accordingly, international consensus statements and the clinical guidelines have endorsed TIR as a key metric for diabetes monitoring, recommending a target of 70% for most non-pregnant adults [15]. Despite its increasing endorsement, the application of TIR in routine decision-making remains limited. A key barrier is the lack of comprehensive and trustworthy quantitative estimates that translate TIR increments into predictable risk reductions for patient-important outcomes, such as cardiovascular or kidney events. To address this gap, we conducted a systematic review and meta-analysis to summarize outcomes related to TIR and to quantitatively estimate its prognostic value across clinically relevant outcomes, thereby facilitating evidence-informed decision-making regarding TIR in clinical practice.

## 2. Research Design and Methods

### 2.1. Literature Search

We systematically searched PubMed, Embase (via OVID), and the Cochrane Central Register of Controlled Trials (CENTRAL, via OVID) from January 2017 to November 2025 using predefined search terms including “time in range” and “target in range”. The comprehensive search strategy is provided in Supplementary Materials Section S2.

### 2.2. Study Selection and Eligibility Criteria

We included studies enrolling adults with type 2 diabetes mellitus (T2DM). Studies involving mixed populations (type 1 and type 2 diabetes) were excluded unless T2DM participants constituted above 80% of the total cohort. The exposure of interest was TIR assessed by CGM, FGM or fingertip capillary glucose monitoring (FCGM). Studies reporting quantitative TIR measurements that allowed dose–response or categorical comparisons were included. To ensure the feasibility of a quantitative synthesis, studies reporting associations solely based on TIR categories (e.g., quantiles) without corresponding mean or continuous TIR values were excluded. We included all reported clinical outcomes, aiming to comprehensively evaluate the associations between TIR and a broad spectrum of clinically relevant endpoints. Peer-reviewed observational studies, including prospective or retrospective cohorts, case–control, and cross-sectional studies were included. We excluded reviews, editorials, conference abstracts with insufficient methodological detail, and studies lacking extractable data.

### 2.3. Study Selection

Two reviewers (FQ and QY) independently screened titles and abstracts after removal of duplicates using Endnote X9 (Version X9.3.3, Clarivate, Philadelphia, PA, USA), followed by full-text assessment for eligibility. Reasons for exclusion were documented and reported. Disagreement was resolved through discussion with a third reviewer (JL). Reference lists of the included literature were manually screened to identify additional studies.

### 2.4. Data Extraction

Data were extracted independently by two investigators using a standardized, predefined data collection form. Extracted information included study characteristics (e.g., author, publication year, study design, sample size, and follow-up duration), participant baseline characteristics (e.g., sample size, age, sex), exposure details (assessment methods for TIR, specific TIR definitions, and mean or median TIR values across groups), and outcomes and effect estimates (clinical outcomes and their most fully adjusted effect estimates with 95% confidence interval [CI]). Detailed definitions of all clinical outcomes are provided in Supplementary Materials Section S5. Any inconsistencies encountered were thoroughly discussed with a senior author (JL).

### 2.5. Data Analysis

All outcomes were rated as critical, important, or not important based on their clinical significance. To align with contemporary clinical standards, albuminuria outcomes from the included studies were reclassified according to the Kidney Disease: Improving Global Outcomes (KDIGO) 2024 Clinical Practice Guideline for the management of chronic kidney disease in diabetes [2]. Specifically, using the urinary albumin-to-creatinine ratio (UACR), “microalbuminuria” was mapped to KDIGO A2 (30–299 mg/g), and “macroalbuminuria” was mapped to KDIGO A3 (UACR ≥300 mg/g). Odd ratios (ORs) were used as the common effect measure to summarize the association between TIR and multiple adverse outcomes. When studies reported hazard ratios (HRs) instead of ORs, we converted HRs to ORs using the square-root transformation method proposed by VanderWeele [16,17] (Supplementary Materials Section S7), which is recommended when the outcome event is relatively common and baseline risk is not directly reported, a scenario that applied to most of the included cohort studies. Detailed conversion procedures are provided in Supplementary Materials Section S8. Given the heterogeneity in the categorization of TIR metrics across studies, effect estimates were standardized to represent a 10% increase in TIR using the most fully adjusted estimates reported in the articles. For studies that reported associations across TIR quantiles, effect estimates were converted to a 10% increase in TIR when the mean TIR values were available (Supplementary Materials Section S6). When the same exposure–outcome association was evaluated across multiple studies, we retained the original outcome definition as reported in each study (Supplementary Materials Section S5). We employed a random-effects model with the generalized inverse-variance method to pool summary effect estimates and their 95% CI [17]. The heterogeneity across studies was assessed using Cochran’s Q-test (with a significance level of *α* = 0.10) and quantified by I^2^ statistic [18]. All statistical analyses were performed using R version 4.2.1 (R Foundation for Statistical Computing, Vienna, Austria) with the ‘meta’ and ‘metafor’ packages. Sensitivity analyses using a fixed-effects model were performed to synthesize the summary effect and leave-one-out analyses to detect the influence of individual studies on the pooled estimates. To assess the robustness of our findings to different glucose monitoring modalities, we performed a sensitivity analysis excluding the three FCGM-based studies, while retaining the FGM-based studies (Abbott Freestyle Libre, Alameda, CA, USA) as they are considered part of the broader CGM family. The results of this analysis are reported in Supplementary Materials Section S13.3. Due to substantial diversity of clinical outcomes and the variability in their definitions across the included studies, a quantitative synthesis was not feasible for all endpoints. Therefore, results were also summarized narratively, complemented by graphic displays and structured summary tables where appropriate.

### 2.6. Quality Assessment

The risk of bias in the included studies was assessed using the Newcastle–Ottawa quality assessment scale (NOS) [19]. The overall certainty of evidence was assessed using the GRADE framework, categorized as high, moderate, low, or very low [20]. For observational studies, the certainty of evidence could be upgraded based on three criteria: (1) large magnitude of effect (OR ≤ 0.9 for a one-level upgrade), (2) evidence of a dose–response gradient, and (3) plausible residual confounding that would bias toward the null. The detailed criteria and operating rules are provided in Supplementary Materials Section S14.

### 2.7. Protocol and Reporting Guidelines

This systematic review was conducted and reported in accordance with the Preferred Reporting Items for Systematic Reviews and Meta-Analyses (PRISMA) 2009 statement [21]. A PRISMA checklist detailing our adherence to the reporting guidelines is provided in the Supplementary Materials (Supplementary Materials Section S1). The review protocol was prospectively registered with the International Prospective Register of Systematic Reviews (PROSPERO) under registration number CRD42022361217.

## 3. Results

### 3.1. Characteristics of Included Studies and Participants

**Figure 1:**
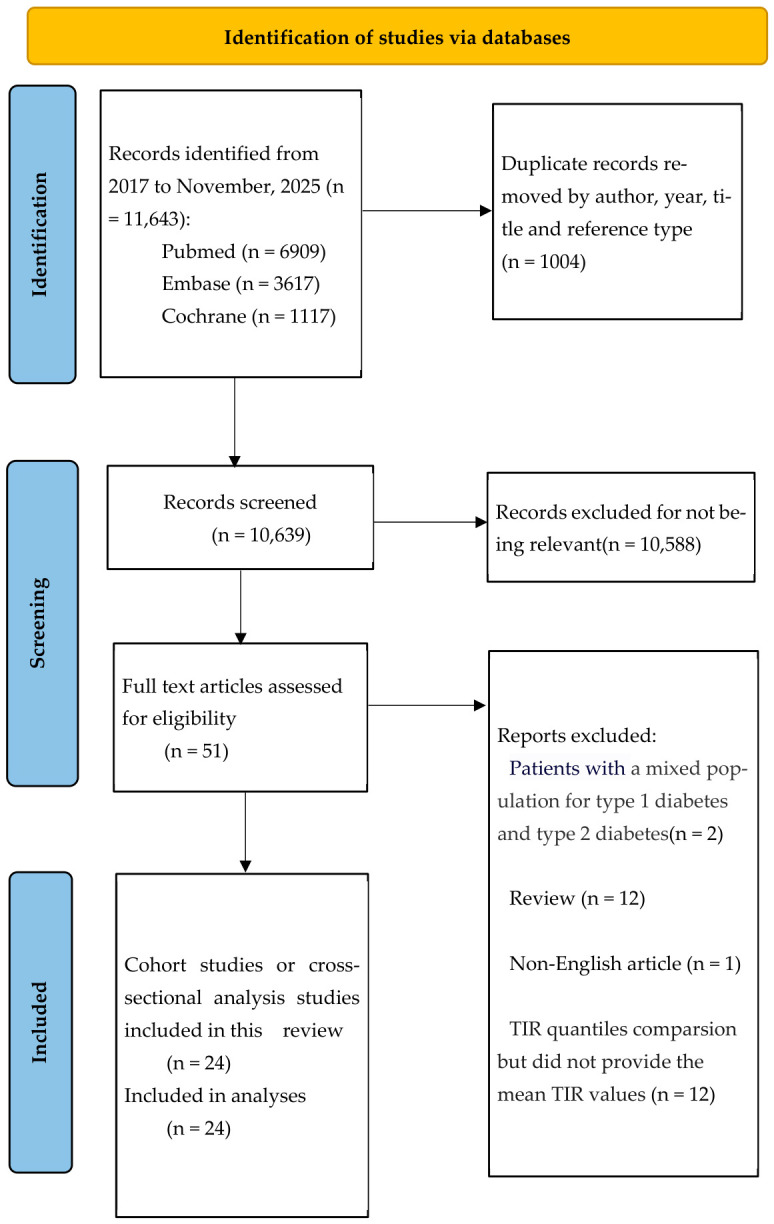
PRISMA flow diagram showing study selection process.

As illustrated in the PRISMA flowchart (Figure 1), our systematic search identified 10,639 records after removal of duplicates. Following title and abstract screening, 51 articles underwent full-text evaluation. Of these, 27 studies were excluded for reasons including mixed study population, review designs, non-English language, or insufficient data for quantitative synthesis (e.g., reporting TIR quantiles without corresponding mean/median values). Ultimately, 24 studies (4 cohort and 20 cross-sectional), comprising a total of 35,916 participants, were included in the final analysis. Although our eligibility criteria permitted the inclusion of mixed-population studies if T2DM participants exceeded 80%, all included studies were exclusively composed of patients with T2DM.

As shown in Table 1, most included studies used Medtronic (Northridge, CA, USA) CGM devices, with a typical measurement duration of 72 h. Regarding statistical methods, five studies employed Cox proportional hazards models to estimate the association between TIR and clinical outcomes, whereas the remaining studies used logistic regression models. Detailed characteristics of each included study are provided in Supplementary Materials Section S3.

**Figure 2:**
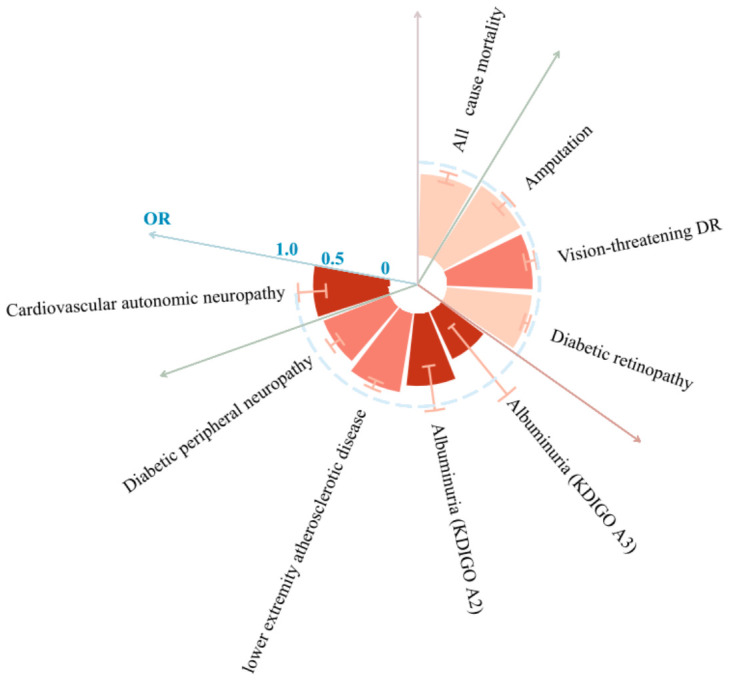
Summary of findings for odds ratio of the association between each 10% rise in TIR and risk of adverse outcomes. Red denotes critical outcomes, orange denotes important outcomes, and yellow denotes not important outcomes. Albuminuria (KDIGO A2) corresponds to microalbuminuria (UACR 30–299 mg/g) and albuminuria (KDIGO A3) to macroalbuminuria (UACR ≥ 300 mg/g) according to KDIGO 2024.

All studies adjusted for age and sex; however, adjustment for additional covariates varied across studies, commonly including body mass index (BMI), lipid profile, duration of diabetes, baseline or mean HbA1c, systolic or diastolic blood pressure, use of statins or aspirin, or glucose-lowering drugs. The detailed adjusted covariates and models for each study are presented in Supplementary Materials Section S10. The quality of the included studies was generally high, with a mean score of 8 based on the NOS. Detailed results of the quality assessment were provided in Supplementary Materials Section S11. Overall, a total of 20 exposure–outcome associations were identified. The detailed effect estimates for all 20 associations are presented in Supplementary Materials Section S4. Of these, nine associations had sufficient data for quantitative meta-analysis; detailed results of all meta-analyses are presented in Supplementary Materials Section S12. The remaining 11 associations were supported by only a single study each and were therefore summarized descriptively (Supplementary Materials Section S9). Forest plots for random-effects and fixed-effects models are provided in Supplementary Materials Section S15 and Supplementary Materials Section S16, respectively. A summary of the pooled effect estimates for all associations is presented in Figure 2. 

### 3.2. Associations Between TIR and Mortality

Every 10% increase in TIR was consistently associated with a reduced risk of mortality across all evaluated categories (Supplementary Materials Section S9). For all-cause mortality, a meta-analysis of three studies showed a 12% risk reduction (OR 0.88; 95% CI: 0.82–0.93). Sensitivity analyses (Supplementary Materials S13), including leave-one-out analysis (Supplementary Materials S13.1) and fixed-effects model analysis (Supplementary Materials S13.2), further demonstrated the robustness of these findings. According to the GRADE framework, the overall certainty of evidence for all-cause mortality was rated as high (Table 2). This rating was based on: (1) a pooled OR of 0.88 meeting the threshold for a one-level upgrade for large effect; (2) a consistent linear dose–response relationship across TIR quantiles in the included studies; and (3) no serious risk of bias, inconsistency, indirectness, or imprecision. For cancer-specific and cardiovascular mortality, data were limited to a single study for each outcome. Therefore, results were summarized descriptively, both of which suggested a potential inverse association between TIR and mortality risk.

### 3.3. Associations Between TIR and Macrovascular Outcomes

For major adverse cardiovascular events, carotid artery vasculopathy, and stroke, only one study was available for each outcome; however, all reported a consistent risk reduction every 10% increment in TIR (Supplementary Materials Section S9). Regarding lower extremity atherosclerotic disease (LEAD), a meta-analysis of three studies demonstrated that each 10% increase in TIR was associated with a 14% risk reduction (OR = 0.86, 95% CI: 0.82–0.91), with no observed heterogeneity (I^2^ = 0%). The GRADE certainty of evidence for LEAD was rated as high. In contrast, a meta-analysis of two studies found no statistically significant association between TIR and amputation risk (OR = 0.95, 95% CI: 0.88–1.03; *p* = 0.084), with moderate heterogeneity (I^2^ = 66.6%) and low GRADE certainty (Table 2).

### 3.4. Associations Between TIR and Microvascular Complications

The association between TIR and several microvascular complications was evaluated, including diabetic peripheral neuropathy (DPN), cardiovascular autonomic neuropathy (CAN), diabetic retinopathy (DR), and albuminuria. For DPN, a meta-analysis of four studies demonstrated that each 10% increase in TIR was associated with a statistically significant 23% risk reduction (OR = 0.77, 95% CI: 0.71–0.84), with no evidence of heterogeneity (I^2^ = 0%) and high certainty of evidence. However, based on a single study, no statistically significant association was observed between TIR and painful diabetic neuropathy (OR = 0.80, 95% CI: 0.50–1.28). For CAN, a meta-analysis of two studies showed that each 10% increase in TIR was associated with a 19% risk reduction (OR = 0.81, 95% CI: 0.68–0.97), despite substantial heterogeneity (I^2^ = 76.5%). The certainty of evidence was rated as moderate. Regarding DR, each 10% increase in TIR was associated with a significantly reduced risk of both vision-threatening DR (OR = 0.93, 95% CI: 0.87–0.98) and overall DR (OR = 0.92, 95% CI: 0.89–0.95).

The GRADE certainty for these outcomes was moderate. Additionally, a single study on mild non-proliferative DR reported a consistent inverse association with TIR (Supplementary Materials Section S9). Both albuminuria types (KDIGO A3 and KDIGO A2) showed an inverse but non-significant trend with TIR (KDIGO A3: OR = 0.58, 95% CI: 0.27–1.21; KDIGO A2: OR = 0.78, 95% CI: 0.58–1.05). Both outcomes were characterized by substantial heterogeneity (I^2^ = 94.8% for KDIGO A3 and 97.2% for KDIGO A2) and low GRADE certainty.

In a sensitivity analysis excluding the FCGM-based study (Supplementary Materials Section S13.3), the heterogeneity for albuminuria (KDIGO A3) was completely eliminated (I^2^ = 0%), with a pooled OR of 0.85 (95% CI: 0.80–0.90). For albuminuria (KDIGO A2), the heterogeneity decreased from 97.2% to 84.6%, with a pooled OR of 0.91 (95% CI: 0.79–1.05). The effect estimate for albuminuria (KDIGO A3) became statistically significant after exclusion, while the estimate for albuminuria (KDIGO A2) remained non-significant but directionally consistent with the primary analysis.

### 3.5. Other Outcomes

Only one study was available for osteoporosis, which reported that a 5% reduction in risk was observed every 10% increment in TIR (OR = 0.95, 95% CI: 0.91–0.98, Supplementary Materials Section S9).

## 4. Discussion

To our knowledge, this is the first systematic review and dose–response meta-analysis to quantify the association between each 10% increase in glucose monitoring-derived TIR and a broad spectrum of clinically relevant outcomes in patients with T2DM. Overall, each 10% increase in TIR was consistently associated with significant risk reductions across multiple outcomes, including all-cause mortality, LEAD, vision-threatening and overall DR, DPN, and CAN, with relative risk reductions ranging from 12% to 23%. In contrast, evidence regarding albuminuria (KDIGO A2 and KDIGO A3) and amputation was inconclusive; the effect estimates did not achieve statistical significance and were derived from lower-certainty evidence. Taken together, these findings establish a compelling inverse association between TIR and adverse diabetes-related outcomes. By providing a quantitative framework, this study supports the utility of TIR as a key prognostic indicator, thereby facilitating evidence-based target setting and clinical decision-making in diabetes management.

The robust association between TIR and adverse diabetes outcomes is supported by well-established pathophysiological mechanisms. Notably, glycemic fluctuations exert a more profound impact on triggering oxidative stress and activating nuclear factor kappa-light-chain-enhancer of activated B cells’ inflammatory cascade than chronic hyperglycemia alone, thereby accelerating endothelial dysfunction. Concurrently, increased hypoglycemic exposure independently precipitates myocardial ischemia and brain injury [22,23,24]. The clinical relevance of these mechanisms is corroborated by our synthesis: among the 24 included studies, 20 (83%) showed that the association between TIR and adverse outcomes remained significant even after adjusting for HbA1c.

While previous studies have suggested that TIR may have greater prognostic value than HbA1c in predicting diabetic-related complications [25,26], existing evidence has been largely derived from individual observational studies or meta-analyses focusing on a single outcome, remaining primarily qualitative in nature. The present study substantially extends this body of evidence. By systematically integrating these observational data into a comprehensive quantitative framework, we calculated standardized effect sizes based on a 10% increment in TIR across multiple complication domains. This rigorous approach definitively confirms that TIR provides incremental prognostic value independent of mean glucose exposure, effectively capturing the distinct clinical burden of “glycemic variability” inherently missed by HbA1c.

Our findings align with and substantially expand upon existing literature. For instance, while a systematic review reported an inverse association between TIR and microvascular complications, their evaluation lacked a dose–response analysis [27]. Similarly, another systematic review consolidated evidence demonstrating that higher TIR was associated with reduced risks of both macrovascular complications, as well as both cardiovascular and all-cause mortality [10]. Building upon these findings, this current study not only quantifies these associations across a broader spectrum of outcomes but uniquely establishes a linear dose–response relationship between TIR and all-cause mortality, DPN, and LEAD, supported by high-certainty GRADE evidence. Nevertheless, the interpretation of these findings requires careful consideration of several methodological factors inherent to the included studies, including CGM duration and device types.

There was considerable variation in CGM duration across studies, ranging from 3 to 14 days, with the majority (54%) employing a 3-day monitoring period. This variability may have prognostic implications, as shorter monitoring periods may be insufficient to adequately capture an individual’s typical glycemic variability and daily patterns, potentially leading to misclassification of TIR and biasing the observed effect estimates towards the null. Studies with longer monitoring periods (e.g., 14 days or two 6-day periods) may provide a more representative assessment of long-term glycemic exposure, although the optimal monitoring duration for predicting outcomes remains to be determined.

Beyond monitoring duration, the type of device used also warrants consideration. The included studies employed diverse devices, including Medtronic CGM (used in 58% of studies), Abbott Freestyle Libre FGM (17%), and Meiqi (Meiqi Medical Systems, Beijing, China) CGM. Notably, the Medtronic iPro device, which was used in a subset of studies, records glucose data blinded to the patient, whereas other devices such as the Freestyle Libre provide real-time feedback. This distinction is clinically relevant, as real-time CGM enables patients to make immediate therapeutic adjustments, potentially yielding greater improvements in glycemic control and clinical outcomes than blinded systems. However, because the effect estimates in our meta-analysis were derived from observational comparisons rather than head-to-head device trials, we could not formally assess whether the prognostic value of TIR differs by device type. Future studies with standardized device protocols are needed to explore this question.

Furthermore, the divergent associations observed between neuropathy/retinopathy and albuminuria may reflect underlying pathophysiological differences. Neuropathy and retinopathy are primarily driven by acute glycemic excursions, which trigger rapid oxidative stress, mitochondrial dysfunction, and inflammatory cascades within neural and retinal microvascular endothelial cells [22,23,24]. In contrast, albuminuria reflects glomerular barrier injury, which is more closely linked to cumulative glycemic burden, hemodynamic alterations (including intraglomerular hypertension), and activation of the renin–angiotensin–aldosterone system [2,3]. Moreover, the kidney’s intrinsic autoregulatory capacity may buffer the impact of short-term glucose fluctuations, rendering albuminuria less sensitive to acute glycemic variability than to chronic hyperglycemic exposure. This mechanistic distinction may explain why the inverse association between TIR and albuminuria was attenuated and more heterogeneous in our meta-analysis, whereas the associations with neuropathy and retinopathy were consistently robust. These findings further support the notion that TIR and HbA1c provide complementary information for risk stratification across different complication types [12,28].

From a clinical perspective, these results provide a much-needed quantifiable frame-work for interpreting changes in TIR in clinical context. The observation that incremental increases in TIR translate to meaningful risk reductions across multiple hard endpoints strongly supports the current international consensus recommending a TIR target of >70% [15]. Crucially, our dose–response data suggest that even incremental improvements below this threshold confer significant prognostic benefit. This continuous benefit model can profoundly aid in clinical interpretation, supporting realistic goal-setting and shared decision-making between clinicians and patients. By communicating that a mere 10% increase in TIR yields a 12% reduction in all-cause mortality, an effect size comparable in magnitude to novel therapeutic agents in cardiovascular outcome trials (CVOTs), clinicians can better motivate patients to achieve incremental improvements [25,28]. However, given the observational nature of the underlying evidence, these findings primarily facilitate risk stratification rather than establishing definitive causal inferences.

Further, for outcomes supported by low-certainty evidence, especially albuminuria and amputation, the observed risk reduction estimates must be interpreted with caution and integrated holistically with other clinical parameters. Regarding albuminuria, the substantial heterogeneity and wide confidence intervals warrant careful consideration; notably, previous studies have reported similar, though not entirely consistent, findings [13,29]. Our sensitivity analysis indicated that the heterogeneity was largely attributable to a single FCGM-based study. After its exclusion, the effect estimate for albuminuria (KDIGO A3) became statistically significant with no residual heterogeneity, whereas the estimate for albuminuria (KDIGO A2) remained non-significant. These findings suggest that methodological differences in glucose monitoring techniques and outcome definitions, specifically the use of FCGM versus CGM and single versus consecutive urine-albumin-to-creatinine ratio (UACR) measurements, may have contributed substantially to the observed inconsistencies. These inconsistencies likely stem from inter-study variability in outcome definitions, follow-up durations, and baseline renal function, underscoring the need for standardized methodologies in future research. Similarly, our non-significant findings regarding amputation align with several previous studies [30,31]. From a clinical perspective, amputation is a complex, multifactorial outcome; its occurrence is influenced not solely by glycemic control, but is heavily influenced by co-existing peripheral arterial disease, severe infections, and the overall quality of diabetic foot management. Additionally, from a statistical standpoint, the limited sample sizes and low incidence of amputation events in existing TIR studies may have resulted in insufficient statistical power, making it difficult to detect a definitive association.

Several limitations of the present study should be acknowledged. First, the included evidence is predominantly derived from observational and cross-sectional studies, with considerable inter-study variability in outcome definitions, glucose monitoring devices, and monitoring durations. These methodological differences limit the ability to establish temporal or causal relationships and may have introduced residual confounding into the pooled estimates. Second, publication bias was not formally assessed using funnel plots or Egger’s test because most meta-analyses included fewer than 10 studies, which renders such tests underpowered and potentially misleading. Third, nearly 80% of the included studies were conducted in China, which may limit the generalizability of our findings to other populations. To address these evidence gaps, future research should prioritize large-scale, multi-center prospective cohorts employing standardized CGM metrics and consistent outcome definitions to better characterize the temporal and potentially causal relationship. Additionally, well-designed randomized controlled trials are warranted to determine whether interventions targeting TIR improvements translate into meaningful reductions in hard clinical endpoints.

## 5. Conclusions

In conclusion, each 10% increase in TIR was significantly associated with reduced risks of multiple adverse outcomes, including all-cause mortality, LEAD, DR, DPN, and CAN, in patients with T2DM, supported by moderate-to-high-certainty evidence. These findings suggest TIR as a robust and clinically meaningful prognostic marker in diabetes management. By quantifying the continuous clinical benefits of glycemic improvement, this study provides an actionable framework that facilitates clinician-patient communication and supports shared decision-making. Ultimately, well-designed prospective studies and randomized controlled trials are warranted to definitively establish whether target interventions to improve TIR translate into tangible reductions in clinical outcomes. However, given the predominantly observational nature of the underlying evidence, causal inferences cannot be established.

## Figures and Tables

**Table 1:** Summary of characteristics of included studies.

Parameter	Summary
Eligible studies	
Total number of trials, *n*	24
Total number of participants, *n*	35,916
Adjusted covariates (number of studies)	
Age	24
Sex	24
BMI	20
TC/HDL/LDL	19
Duration of diabetes	19
Mean HbA1c	5
Baseline HbA1c	15
Baseline systolic/diastolic pressure	15
Baseline drug treatment for diabetes	10
Baseline use statins/aspirin	8
Regression model	
Cox proportional hazard model	5
Logistic regression model	19
Region	
China, *n* (%)	19 (79)
USA, *n* (%)	2 (8)
Japan, *n* (%)	1 (4)
Korea, *n* (%)	2 (8)
Device used	
Medtronic, *n* (%)	14 (58)
Abbott Freestyle Libre, *n* (%)	4 (17)
Meiqi, *n* (%)	3 (12.5)
FCGM, *n* (%)	3 (12.5)
Duration of device use	
3 days, *n* (%)	13 (54)
5 days, *n* (%)	1 (4)
7 days, *n* (%)	2 (8)
14 days, *n* (%)	3 (12.5)
Two 6-day periods separated by 2 weeks, *n* (%)	1 (4)
3 and 6 days for GOLD (Medtronic) and iPro2 (Medtronic), respectively, *n* (%)	2 (8)

TC, total cholesterol; HDL, high-density lipoprotein; LDL, low-density lipoprotein.

**Table 2:** GRADE assessment for meta-analyses of observational studies.

Outcome	Number of Studies	Cases/Total	OR(95% CI) of 10% TIR Increase	*p*	I^2^ (%)	GRADE Assessment
All-cause mortality	3	1179/7802	0.88 (0.82–0.93)	0.049	66.9%	High ^e,f^
Amputation	2	70/358	0.95 (0.88–1.03)	0.084	66.6%	Low ^a,f^
Vision-threatening DR	2	266/4261	0.93 (0.87–0.98)	0.365	0%	Moderate ^f^
Diabetic retinopathy	3	1476/5590	0.92 (0.89–0.95)	0.171	43.4%	Moderate ^f^
Albuminuria (KDIGO A3)	3	603/3287	0.58 (0.27–1.21)	<0.0001	94.8%	Low ^b,d,e,f^
Albuminuria (KDIGO A2)	3	1160/2879	0.78 (0.58–1.05)	<0.0001	97.2%	Low ^a,d,e,f^
Lower extremity atherosclerotic disease	2	511/1687	0.86 (0.82–0.91)	0.345	0%	High ^e,f^
Diabetic peripheral neuropathy	4	303/1517	0.77 (0.71–0.84)	0.508	0%	High ^e,f^
Cardiovascular autonomic neuropathy	2	205/633	0.81 (0.68–0.97)	0.039	76.5%	Moderate ^c,e,f^

Downgrade reason of GRADE assessment: ^a^ serious imprecision: CI overlaps no effect; ^b^ very serious imprecision: CI overlaps no effect and wide CIs; ^c^ serious inconsistency: a significant test for heterogeneity and high I^2^; ^d^ very serious inconsistency: wide variation in point estimates and appreciable nonoverlap in CIs, a significant test for heterogeneity and high I^2^. Upgrade reason of GRADE assessment: ^e^ large effect size: OR < 0.9; ^f^ dose–response gradient: linearity of the dose–response relationship between TIR and adverse outcomes. Albuminuria (KDIGO A2) corresponds to microalbuminuria (UACR 30–299 mg/g) and albuminuria (KDIGO A3) to macroalbuminuria (UACR ≥ 300 mg/g) according to KDIGO 2024.

## Data Availability

No new data were created or analyzed in this study. Data sharing is not applicable to this article.

## Supplementary Materials

The following supporting information can be downloaded at https://www.mdpi.com/article/10.3390/jcm15145713/s1: Supplementary Materials Section S1: PRISMA 2009 Checklist; Supplementary Materials Section S2: Search strategy; Supplementary Materials Section S3: Characteristics of included studies; Supplementary Materials Section S4: Summary findings for TIR factor and effect sizes for diabetic complications; Supplementary Materials Section S5: Definitions of outcomes; Supplementary Materials Section S6: 10% rise in TIR comparison converted by TIR quantiles; Supplementary Materials Section S7: Studies involved in effect conversion from HR to OR; Supplementary Materials Section S8: Methods of HRs converted to ORs; Supplementary Materials Section S9: Summary effect of association between TIR and adverse diabetes-related outcomes; Supplementary Materials Section S10: Adjusted covariates and adjusted model; Supplementary Materials Section S11: NOS assessments; Supplementary Materials Section S12: Meta-analyses of observational studies; Supplementary Materials Section S13: Sensitivity analyses(including S13.1 Leave-one-out analysis, S13.2 Fixed-effect model analysis, and S13.3 Sensitivity analysis excluding FCGM-based studies); Supplementary Materials Section S14: GRADE assessment criteria; Supplementary Materials Section S15: Random-effects model analysis forest plots; Supplementary Materials Section S16: Fixed-effects model analysis forest plots.
