# Supplementary material for: Time in Range and Adverse Outcomes in Type 2 Diabetes: A Quantitative Synthesis
Source: J Clin Med. 2026 Jul 21;15(14):5713. doi: 10.3390/jcm15145713 (PMC13413012; doi:10.3390/jcm15145713)
Supplement: Supplementary file 1 [file jcm-15-05713-s001.zip › jcm-4380581-supplementary.pdf]

Supplementary Materials Table of Contents

S1. PRISMA 2009 Checklist....2
S2. Search strategy....5
S3. List of characteristics of included studies....6
S4. Summary findings for TIR factor and effect sizes for diabetic complications....9
S5. Definition of outcomes. 13
S6. 10% raise of TIR comparison converted by TIR quantiles....17
S7. The studies involved in effect conversion from HR to OR....18
S8. Methods of the HRs converted to ORs for the studies that did not report the ORs....19
S9. The summary effect of association between TIR and adverse diabetes-related outcomes....20
S10. Adjusted covariates and adjusted model....23
S11. NOS assessments....26
S12. Meta-analyses of observational studies....28
S13. Sensitivity analyses....30
13.1 Leave-one-out analysis....30
13.2 fixed effect model analysis....33
13.3 Sensitivity analysis excluding FCGM-based studies....34
S14. Grade assessment criteria for Meta-analyses of observational studies. 35
S15. Random effect model analysis forest plots....37
S16. Fixed effect model analysis forest plots....40

S1. PRISMA 2009 Checklist

Section/topic	#	Checklist item	Reported on page #
**TITLE**			
Title	1	Identify the report as a systematic review, meta-analysis, or both.	1
**ABSTRACT**			
Structured summary	2	Provide a structured summary including, as applicable: background; objectives; data sources; study eligibility criteria, participants, and interventions; study appraisal and synthesis methods; results; limitations; conclusions and implications of key findings; systematic review registration number.	1
**INTRODUCTION**			
Rationale	3	Describe the rationale for the review in the context of what is already known.	2
Objectives	4	Provide an explicit statement of questions being addressed with reference to participants, interventions, comparisons, outcomes, and study design (PICOS).	2
**METHODS**			
Protocol and registration	5	Indicate if a review protocol exists, if and where it can be accessed (e.g., Web address), and, if available, provide registration information including registration number.	2–4
Eligibility criteria	6	Specify study characteristics (e.g., PICOS, length of follow-up) and report characteristics (e.g., years considered, language, publication status) used as criteria for eligibility, giving rationale.	2–4
Information sources	7	Describe all information sources (e.g., databases with dates of coverage, contact with study authors to identify additional studies) in the search and date last searched.	2–4
Search	8	Present full electronic search strategy for at least one database, including any limits used, such that it could be repeated.	2–4
Study selection	9	State the process for selecting studies (i.e., screening, eligibility, included in systematic review, and, if applicable, included in the meta-analysis).	2–4
Data collection process	10	Describe method of data extraction from reports (e.g., piloted forms, independently, in duplicate) and any processes for obtaining and confirming data from investigators.	2–4
Data items	11	List and define all variables for which data were sought (e.g., PICOS, funding sources) and any assumptions and simplifications made.	2–4

Risk of bias in individual studies	12	Describe methods used for assessing risk of bias of individual studies (including specification of whether this was done at the study or outcome level), and how this information is to be used in any data synthesis.	2–4
Summary measures	13	State the principal summary measures (e.g., risk ratio, difference in means).	2–4
Synthesis of results	14	Describe the methods of handling data and combining results of studies, if done, including measures of consistency (e.g., I^2) for each meta-analysis.	2–4
**Section/topic**	**#**	**Checklist item**	**Reported on page #**
Risk of bias across studies	15	Specify any assessment of risk of bias that may affect the cumulative evidence (e.g., publication bias, selective reporting within studies).	2–4
Additional analyses	16	Describe methods of additional analyses (e.g., sensitivity or subgroup analyses, meta-regression), if done, indicating which were pre-specified.	2–4
**RESULTS**			
Study selection	17	Give numbers of studies screened, assessed for eligibility, and included in the review, with reasons for exclusions at each stage, ideally with a flow diagram.	4–9
Study characteristics	18	For each study, present characteristics for which data were extracted (e.g., study size, PICOS, follow-up period) and provide the citations.	4–9
Risk of bias within studies	19	Present data on risk of bias of each study and, if available, any outcome level assessment (see item 12).	4–9
Results of individual studies	20	For all outcomes considered (benefits or harms), present, for each study: (a) simple summary data for each intervention group (b) effect estimates and confidence intervals, ideally with a forest plot.	4–9
Synthesis of results	21	Present results of each meta-analysis done, including confidence intervals and measures of consistency.	4–9
Risk of bias across studies	22	Present results of any assessment of risk of bias across studies (see Item 15).	4–9
Additional analysis	23	Give results of additional analyses, if done (e.g., sensitivity or subgroup analyses, meta-regression [see Item 16]).	4–9
**DISCUSSION**			
Summary of evidence	24	Summarize the main findings including the strength of evidence for each main outcome; consider their relevance to key groups (e.g., healthcare providers, users, and policy makers).	9–12
Limitations	25	Discuss limitations at study and outcome level (e.g., risk of bias), and at review-level (e.g., incomplete retrieval of identified research, reporting bias).	9–12
Conclusions	26	Provide a general interpretation of the results in the context of other evidence, and implications for future research.	12
**FUNDING**			

Funding	27	Describe sources of funding for the systematic review and other support (e.g., supply of data); role of funders for the systematic review.	13

From: Moher D, Liberati A, Tetzlaff J, Altman DG, The PRISMA Group (2009). Preferred Reporting Items for Systematic Reviews and Meta-Analyses: The PRISMA Statement. PLoS Med 6(6): e1000097. doi:10.1371/journal.pmed1000097

S2. Search strategy

Databases: PubMed, Embase and Cochrane Library from 2017 to November 2025.

Search Terms:

1. TIR. ab, ti.

2. Time in range. ab, ti.

3. Time-in-range. ab, ti.

4. 1 or 2 or 3

5. Target in range. ab, ti.

6. Glucose. ab, ti.

7. Glycemic. ab, ti.

8. Glycaemic. ab, ti.

9. 5 and (6 or 7 or 8)

10. 4 or 9

("TIR" OR "Time in range" OR "Time-in-range") OR (("Target in range" AND ("Glucose" OR "Glycemic"OR "Glycaemic"))

S3. List of characteristics of included studies.

Author/ Year	Country	Age(Mean)	Male(%)	BMI (kg/ m2)	Baseline mean HbA1c, %	Duration of diabetes(years)	Insulin(%)	Observation time(median/mean)	Duration of glucose monitoring use	glucose monitoring device used	Study designs
Li,F.2020	China	60.2	51.0	22.1	8.5	10.7	-	April 2013 and August 2014	3 days	Medtronic	Cross-sectional analysis
Guo,Q.Y.2020	China	54.5	70.0	25.5	8.4	7.9	14.1	October 2017 to May 2019	3 days	Meiqi	Cross-sectional analysis
Yoo,J.H. 2020	Korea	58.5	60.5	25.1	8.2	13.1	52.4	March 2009 to May 2019	3 and 6 days for GOLD (Medtronic)and iPro2 (Medtronic),respectively	Medtronic	Cross-sectional analysis
Li,J.2020	China	60.4	56.4	24.6	8.4	8.5	40.1	January 2018 and July 2020	3 days	Abbott FreeStyle Libre	Cross-sectional analysis
Lu,J.2020	China	60.4	53.5	20.1	9.0	8.5	68.1	June 2006 – June 2011	3 days	Medtronic	Cross-sectional analysis
Lu,J.2021	China	61.7	54.7	24.9	8.9	9.7	66.9	January 2005 to December 2015	3 days	Medtronic	Cohort study
Lu,J.2018	China	60.4	44.7	25.1	8.9	8.1	68.4	January 2005 to the end of February 2012	3 days	Medtronic	Cross-sectional analysis

Guo,J.2021	China	69.8	49.1	26.4	7.9	-	-	April 2013 to June 2017	3 days	Medtronic	Cross-sectional analysis
Yang,J.2021	China	53.0	67.3	24.8	7.4	8.1	14.5	July 2018 to May 2019	14 days	Abbott FreeStyle Libre	Cross-sectional analysis
Kim,M.Y.2021	Korea	57.3	58.4	24.8	8.2	12.1	48.1	March 2009 to July 2019	3 and 6 days for GOLD (Medtronic)and iPro2 (Medtronic),respectively	Medtronic	Cross-sectional analysis
Guo,Q.2020	China	52.4	65.3	25.1	9.2	6.4	24.3	April 2018 to August 2019	3 days	Meiqi	Cross-sectional analysis
Huang,R.2022	China	63.0	50.0	24.6	8.1	8.5	14.9	Jan 2019 to May 2020	3 days	Medtronic	Cross-sectional analysis
Chai,S.2022	China	55.6	62.8	26.0	8.4	8.9	49.0	January 2018 to December 2019	-	Fingertip capillary blood glucose monitoring (FCGM)	Cross-sectional analysis
Yin,X.2022	China	68.3	38.0	25.5	7.9	6.0	-	March to December in 2019	5 days	Medtronic	Cross-sectional analysis
Wang,Y.2022	China	61.0	62.0	25.0	8.4	12.0	72.3	May 2020 to October 2021	7 days	Medtronic	Cross-sectional analysis
Wang,Y.2022(2)	China	58.0	56.6	25.0	8.4	9.0	59.7	April 2005 to May 2019	3 days	Medtronic	Cohort study

Shen,Y. 2022	China	61.5	51.0	24.9	8.9	9.6	85.4	January 2005 to December 2015	3 days	Medtronic	Cohort study
Xie,P.2022	China	66.0	55.4	29.4	8.6	9.7	-	January 2009 and June 2014	14 days	Fingertip capillary blood glucose monitoring (FCGM)	Cross-sectional analysis
Zhao,W. 2022	China	61.0	58.7	24.8	8.2	11.0	-	September 2020 to July 2021	7 days	Medtronic	Cross-sectional analysis
Mayeda, L.2020	USA	68.0	63.8	33.8	7.8	20.0	88.6	August 2015 to July 2017	Two 6-day periods, separated by 2 weeks	Medtronic	Cross-sectional analysis
Wakasugi,S.2021	Japan	64.6	60.9	24.6	7.1	12.9	15.8	May 2018 and March 2019	14 days	Abbott FreeStyle Libre	Cross-sectional analysis
Richard, B.2023	USA	65.0	62.4	33.6	8.4	16.4	-	1.99 years (minimum 0.00 years; maximum 2.75 years)	-	Fingertip capillary blood glucose monitoring (FCGM)	Cross-sectional analysis
C. Xu.2025	China	57.8	57.0	24.8	8.0	7.6	51.0	01/09/2020 to 30/06/2023	3 days	Meiqi	Cross-sectional analysis
Q. Zhang.2025	China	61.0	58.2	23.8	8.6	5.0	41.4	July 2020 to December 2022	3 days	Abbott Japan	Cohort study

S4. Summary findings for TIR factor and effect sizes for diabetic complications.

Outcome/Study	Effect size(95%CI)		
	Each 10% raise of TIR	Quantile-3 of TIR	Quantile-4 of TIR
**Mortality**			
**Xie,P.2022**	All-cause mortality:OR0.88 (0.78–0.98)	All-cause mortality:OR0.04 (0.00-1.60)	–
**Shen,Y.2022**	Cancer mortality:HR0.93 (0.87–0.99)	-	-
**Lu,J.2021**	All-cause mortality:HR0.92 (0.89–0.96)<br>Cardiovascular mortality:HR0.95 (0.90–1.00)	-	All-cause mortality:HR0.55 (0.44-0.68)<br>Cardiovascular mortality:HR0.54 (0.37-0.80)
**Q. Zhang.2025**	All-cause mortality:HR:0.86 (0.81;0.92)	All-cause mortality:HR:0.53 (0.37;0.75)	
**Abnormal carotid intima-media thickness (CIMT)/Carotid artery-max-intima-media thickness-change**			
**Lu,J.2020**	Abnormal CIMT:OR0.936 (0.878-0.998)		
**Major adverse cardiovascular events(MACE)**			
**Richard, B.2023**	MACE:HR0.94 (0.90–0.98)		

**Stroke**			
**Guo,J.2021**	OR0.89 (0.82,0.95)		OR0.59 (0.50,0.74)
**Diabetic nephropathy(DN)**			
**Yoo,J.H.2020**	KDIGO A2:OR0.97(0.95-1.04)		
**Chai,S.2022**	KDIGO A2:OR0.58 (0.52,0.65)<br>KDIGO A3:OR0.26 (0.18,0.38)		
**Wakasugi,S.2021**	KDIGO A3:OR0.81 (0.72,0.90)		
**Q. Zhang.2025**	KDIGO A3:HR0.91 (0.86,0.96)	KDIGO A3:HR0.72 (0.54;0.96)	
**Amputation/lower extremity atherosclerotic disease (LEAD)**			
**Xie,P.2022**	Amputation:OR0.98 (0.96–0.99)		Amputation:OR0.18 (0.04–0.79)
**Li,J.2020$**	Presence LEAD:OR0.81 (0.70-0.92)		
**Yin,X.2022#**	Amputation:OR0.90 (0.81–0.98)		
**Wang,Y.2022(2)**	Incident LEAD:HR0.92 (0.87–0.97)<br>Progressive LEAD:HR0.92 (0.83–1.01)		Incident LEAD:HR0.59 (0.43-0.83)<br>Progressive LEAD:HR0.63 (0.33-1.18)
**Diabetic retinopathy(DR)**			
**Lu,J.2018**	Vision-threatening DR:OR0.91 (0.85–0.98)<br>Moderate nonproliferative DR (NPDR):OR0.91 (0.84–0.98)<br>DR:OR0.92 (0.88–0.96)<br>Mild nonproliferative DR		Vision-threatening DR:OR0.52(0.28–0.95)<br>Moderate nonproliferative DR:OR0.40 (0.22–0.73)<br>DR:OR0.50 (0.35–0.71)<br>Mild nonproliferative DR:OR0.56

	(NPDR):OR0.93 (0.87–0.99)		(0.34–0.90)
**Wang,Y.2022**	DR:OR0.93 (0.88-0.97)		DR:OR0.73 (0.54-0.99)
**Wakasugi,S.2021**	DR severity (NDR/SDR/PPDR/PDR):OR0.97 (0.86-1.09)		
**C. Xu.2025**	DR:OR0.53 (0.29-0.94)		DR:OR2.33(0.73-7.44)
**Diabetic peripheral neuropathy(DPN)/Painful diabetic neuropathy(PDN)/Diabetic sensorimotor polyneuropathy(DSPN)**			
**Li,F.2020**		DPN:OR0.41 (0.25–0.67)	
**Guo,Q.Y.2020**[$]	DPN:OR0.78 (0.71-0.87)	DPN:OR0.32 (0.12-0.83)	
**Yang,J.2021**			Prevalence of PDN (Moderate/severe pain):OR0.19 (0.05-0.76)<br>Prevalence of PDN (Mild pain):OR0.37 (0.12-1.12)<br>Prevalence of PDN (Any pain):OR0.35 (0.12-0.98)
**Mayeda,L.2020**	DPN:OR0.81 (0.66-1.01)		
**Zhao,W.2022**	Early nerve damage of DSPN:OR0.67(0.54-0.84)		
**Cardiovascular autonomic neuropathy (CAN)**			
**Kim,M.Y.2021**	Presence of CAN:OR0.89 (0.79–1.00)		
**Guo,Q.2020**[$]	Severe CAN:OR0.56 (0.39-0.78)<br>Any CAN:OR0.74 (0.64-0.83)		CAN:OR0.14 (0.04-0.46)

Osteoporosis	
**Huang,R.2022**[$]	Osteoporosis:OR0.95 (0.91-0.98)

Footnotes: Red represent critical outcomes; orange represent important outcomes; yellow represent not important outcomes. [$]effect value 10% raise of TIR converted by per 1% raise of TIR；[#]effect value 10% raise of TIR converted by per 5% raise of TIR.

S5. Definition of outcomes.

Study ID	Outcome	Definition
**TIR:each 10% raise**		
**Xie,P.2022**	All-cause mortality	All-cause mortality
**Lu,J.2021**	All-cause mortality	All-cause mortality
**Q. Zhang.2025**	All-cause mortality	All-cause mortality
**Shen,Y.2022**	Cancer mortality	The death causes were identfied with the use of the codes in the International Classification of Disease 10th version (ICD-10), ICD-10 codes C00 through C99 were classified as death of cancer.
**Lu,J.2021**	Cardiovascular mortality	The death causes were identified with the use of the codes in the ICD10. ICD codes I00 through I99 were classified as CVD deaths.
**Richard, B.2023**	MACE	Major adverse cardiovascular events(death from cardiovascular causes, nonfatal myocardial infarction, or nonfatal stroke).
**Guo,J.2021**	Stroke	Stroke
**Xie,P.2022**	Amputation	303 enrolled patients, 50 (16.5%) had undergone amputation whereas seven (2.3%) were deceased.
**Yin,X.2022**	Amputation	55 patients with DFO, all of whom underwent any amputation from March to December in 2019.
**Lu,J.2018**	Vision-threatening DR	Proliferative DR was combined with severe nonproliferative DR; together these were defined as vision-threatening DR (VTDR).
**Lu,J.2018**	Moderate nonproliferative DR	Retinopathy was graded according to the International Classification of Diabetic Retinopathy (11). The severity of DR was classified as 1) non-DR (NDR); 2) mild nonproliferative DR (NPDR); 3) moderate NPDR; 4) severe NPDR; and 5) proliferative DR (PDR)
**Lu,J.2018**	Diabetic retinopathy	The presence of any DR (according to the International Classification of Diabetic Retinopathy)

**Wang,Y.2022**	Diabetic retinopathy	DR was diagnosed and graded by severity according to the International Classification of Diabetic Retinopathy
**Wakasugi,S.2021**	Diabetic retinopathy severity (NDR/SDR/PPDR/PDR)	The patients were grouped into four groups based on medical records: no diabetic retinopathy (NDR), simple diabetic retinopathy (SDR), preproliferative diabetic retinopathy (PPDR), or proliferative diabetic retinopathy (PDR).
**C. Xu.2025**	Diabetic retinopathy	According to fundus photography, participants were divided into DR group (DR, 97 cases) and non-DR group (NDR, 201 cases)
**Wakasugi,S.2022**	Albuminuria severity (normol/KDIGO A2/KDIGO A3)	DN was defined according to the level of UAE: <30 mg/g creatinine was defined as normoalbuminuria, 30–299 mg/g creatinine was defined as microalbuminuria, and ≥300 mg/g creatinine was defined as macroalbuminuria.
**Chai,S.2022**	Albuminuria (KDIGO A3)	Macroalbuminuria (levels >300 mg/g).
**Q. Zhang.2025**	Albuminuria (KDIGO A3)	Macroalbuminuria (levels >300 mg/g).
**Yang,J.2021**	Moderate to severe pain of painful diabetic neuropathy	The DPN participants were asked to record any form of pain, such as a burning sensation; feeling of electric shocks, or a stabbing sensation in the toes, feet or legs every day during the 2-week monitoring period. If the pain became unbearable and required medication, the observation was terminated at any time.The severity of neuropathic pain was quantified using an 11step NRS After the observation period, the mean level of NRS was calculated, and the definition and severity of pain were rated according to the average NRS, with a score of 0 indicating no pain. PDN was defined as an NRS score of ≥1:1–3 indicated mild pain and 4–10 indicated moderate/severe pain.
**Guo,Q.2020**	Severe cardiovascular autonomic neuropathy	Minimum and maximum score were 0 and 4, separately. The CAN score0-0.5 and 1-1.5 was considered absent and early CAN,respectively. The CAN score ≥ 2 with or without orthostatic hypotension was considered severe and definite CAN.
**Huang,R.2022**	Osteoporosis	T-score ≥ −1.0 indicated normal, osteopenia was defined by -2.5 < T-score < −1.0, and osteoporosiswas diagnosed as a T-score ≤ −2.5.
**Chai,S.2022**	Albuminuria (KDIGO A2)	Microalbuminuria (levels of 30–300 mg/g)
**Yoo,J.H.2020**	Albuminuria (KDIGO	Microalbuminuria (levels of 30–300 mg/g)

	A2)	
**Lu,J.2018**	Mild nonproliferative diabetic retinopathy (NPDR)	Retinopathy was graded according to the International Classification of Diabetic Retinopathy (11). The severity of DR was classified as 1) non-DR (NDR); 2) mild nonproliferative DR (NPDR); 3) moderate NPDR; 4) severe NPDR; and 5) proliferative DR (PDR)
**Lu,J.2020**	Abnormal carotid intima-media thickness (CIMT)	The common carotid artery IMT was measured on-line in the posterior wall 10–20 mm proximal to the carotid bifurcation in a region free of focal plaque. Three measurements were made on each side, and the values were averaged to produce a mean IMT for each side. The mean CIMT was calculated as the mean of the right and left IMT of the common carotid artery. Abnormal CIMT was defined as a mean CIMT ≥ 1.0mm
**Li,J.2020**	Lower extremity atherosclerotic disease	Patients diagnosed as LEAD had limb extremity arteries examined by the vivid 7 imaging color doppler diagnostic apparatus.
**Wang,Y.2022(2)**	lower extremity atherosclerotic disease	Incident LEAD was defined as the first occurrence of LEAD during follow-up in participants without LEAD at baseline
**Li,F.2020**	Diabetic peripheral neuropathy	CV, latency, and amplitude value was converted into Z-scores as previously described
**Mayeda,L.2020**	Diabetic peripheral neuropathy	Neuropathy Screening Instrument (MNSI) questionnaire to assess symptoms of peripheral neuropathy
**Guo,Q.Y.2020**	Diabetic peripheral neuropathy	The measurements of SUDOSCAN including ESC (measured in μS) and symmetry ratio in hands (HASYM) and feet (FASYM). In our study, we applied 60 μS of average FESC as the diagnostic threshold for sudomotor dysfunction on the basis of previous research. Average FESC ≥ 60 μS was regarded as sudomotor dysfunction (-)
**Zhao,W.2022**	Early nerve damage of diabetic sensorimotor polyneuropathy (DSPN)	Any pain,numbness,tingling,foot weakness,ataxia, or upper-limb symptoms.
**Yang,J.2021**	Mild pain of painful diabetic neuropathy	The DPN participants were asked to record any form of pain, such as a burning sensation; feeling of electric shocks, or a stabbing sensation in the toes, feet or legs every day during the 2-week monitoring period. If the pain became unbearable and required medication, the observation was terminated at any time.The severity of neuropathic pain was quantified using an 11step NRS After the observation period, the mean level of NRS was calculated, and the definition and severity of pain were

		rated according to the average NRS, with a score of 0 indicating no pain. PDN was defined as an NRS score of ≥1:1–3 indicated mild pain and 4–10 indicated moderate/severe pain.
**Guo,Q.2020**	Cardiovascular autonomic neuropathy	Minimum and maximum score were 0 and 4, separately. The CAN score0-0.5 and 1-1.5 was considered absent and early CAN,respectively. The CAN score ≥ 2 with or without orthostatic hypotension was considered severe and definite CAN.
**Kim,M.Y.2021**	Cardiovascular autonomic neuropathy (CAN)	CAN was defined as an abnormal result in two or more parasympathetic test.

S6. 10% raise of TIR comparison converted by TIR quantiles.

Outcomes	Studies	Quantile	Metrics	Effect size of quantile(95%CI)	Effect size of 10% raise(95%CI)
Moderate to severe pain of painful diabetic neuropathy	Yang,J.2021	Quantile4	OR	0.19 (0.05-0.76)	0.69 (0.10-1.28)
Diabetic peripheral neuropathy (DPN)	Li,F.2020	Quantile3	OR	0.41 (0.25–0.67)	0.86 (0.64–1.08)
Mild pain of painful diabetic neuropathy	Yang,J.2021	Quantile4	OR	0.37 (0.12-1.12)	0.80 (0.50-1.28)

S7. The studies involved in effect conversion from HR to OR

Outcomes	Studies	Study designs	HR(95%CI)	OR(95%CI)
All-cause mortality	**Lu,J.2021**	cross-sectional analysis	0.92 (0.89–0.96)	0.91 (0.88–0.94)
All-cause mortality	**Q. Zhang.2025**	cohort study	0.86 (0.81;0.92)	0.83 (0.78-0.89)
Cancer mortality	**Shen,Y.2022**	cross-sectional analysis	0.93 (0.87–0.99)	0.89 (0.85–0.97)
Cardiovascular mortality	**Lu,J.2021**	cohort study	0.95 (0.90–1.00)	0.94 (0.89–1.00)
Major adverse cardiovascular events	**Richard, B.2023**	cross-sectional analysis	0.94 (0.90–0.98)	0.92 (0.88-0.96)
Albuminuria (KDIGO A3)	**Q. Zhang.2025**	cohort study	0.91 (0.86,0.96)	0.86 (0.81-0.92)
lower extremity atherosclerotic disease	**Wang,Y.2022 (2)**	cohort study	0.92 (0.87–0.97)	0.87 (0.82–0.92)

S8. Methods of the HRs converted to ORs for the studies that did not report the ORs

In this review, some included studies did not use odds ratios (OR) to measure the effect but instead of using hazard ratios (HRs). Since they all represent the relative effect, we converted the HRs to ORs and pooled them together.

Scenario 1
When the study reported baseline risk—the risk or incidence if the population was untreated, we can directly use the formula to calculate the ORs from RRs and baseline risk (p0).

$$RR = \frac{OR}{(1 - p_0 + (OR * p_0))}$$

If the study reported HRs, one can convert HRs to RRs firstly.

$$HR = \frac{\log(1 - RR * p_0)}{\log(1 - p_0)}$$

Scenario 2
When the study did not report the baseline risk, we can approximately calculate the ORs from RRs.

$$RR = \sqrt{OR}$$

When the study reported HR, the RR was approximately calculated by the square-root transformation.

$$RR \approx (1 - 0.5^{\sqrt{HR}})/(1 - 0.5^{\sqrt{1/HR}})$$

Reference:

Zhang, J., & Yu, K. F. (1998). What's the relative risk? A method of correcting the odds ratio in cohort studies of common outcomes. JAMA, 280(19), 1690–1691.
VanderWeele T. J. (2017). On a Square-Root Transformation of the Odds Ratio for a Common Outcome. Epidemiology (Cambridge, Mass.), 28(6), e58–e60.
VanderWeele T. J. (2020). Optimal approximate conversions of odds ratios and hazard ratios to risk ratios. Biometrics, 76(3), 746–7

S9. The summary effect of association between TIR and adverse diabetes-related outcomes.

Outcome	Study ID	Study designs	Metrics	Number of participants	Narrative summary Effect estimate (95% CI)	Merged effect estimate (95% CI)
**TIR:Each 10% raise**						
All-cause mortality	**Xie,P.2022**	cross-sectional analysis	OR	303	0.88 (0.78–0.98)	0.88 (0.82-0.93)
	**Lu,J.2021**	cross-sectional analysis	OR*	6225	0.91(0.88–0.94)	
	**Q. Zhang.2025**	cohort study	OR*	1274	0.83 (0.78-0.89)	
Cardiovascular mortality	**Lu,J.2021**	cohort study	OR*	6225	0.94 (0.89–0.99)	
Cancer mortality	**Shen,Y.2022**	cross-sectional analysis	OR*	6225	0.89 (0.85–0.93)	
Major adverse cardiovascular events	**Richard, B.2023**	cross-sectional analysis	OR*	5774	0.92 (0.88-0.96)	
Stroke	**Guo,J.2021**	cross-sectional analysis	OR	510	0.89 (0.82-0.95)	
Amputation	**Xie,P.2022**	cross-sectional analysis	OR	303	0.98 (0.96–0.99)	0.95 (0.88-1.03)
	**Yin,X.2022**	cross-sectional analysis	OR	55	0.90 (0.81–0.98)	
Vision-threatening DR	**Lu,J.2018**	cross-sectional analysis	OR	3262	0.91 (0.85–0.98)	0.93 (0.87-0.98)
	**Wakasugi,S.2021**	cross-sectional analysis	OR	999	0.97 (0.86-1.09)	
Moderate nonproliferative DR	**Lu,J.2018**	cross-sectional analysis	OR	3262	0.91 (0.84–0.98)	
Diabetic retinopathy	**Lu,J.2018**	cross-sectional analysis	OR	3262	0.92 (0.88–0.96)	0.92 (0.89–0.95)
	**Wang,Y.2022**	cross-sectional analysis	OR	2030	0.93 (0.88-0.97)	
	**C. Xu.2025**	cohort study	OR	298	0.53 (0.29,0.94)	

Albuminuria (KDIGO A3)	**Wakasugi,S.2022**	cross-sectional analysis	OR	999	0.81 (0.72-0.90)	0.58 (0.27-1.21)
	**Chai,S.2022**	cross-sectional analysis	OR	1014	0.26 (0.18,0.38)	
	**Q. Zhang.2025**	cohort study	OR*	1274	0.86 (0.81-0.92)	
Moderate to severe pain of painful diabetic neuropathy	**Yang,J.2021#**	cross-sectional analysis	OR	364	0.69 (0.10-1.28)	
Severe cardiovascular autonomic neuropathy	**Guo,Q.2020**	cross-sectional analysis	OR	349	0.56 (0.39-0.78)	
Osteoporosis	**Huang,R.2022**	cross-sectional analysis	OR	362	0.95 (0.91-0.98)	
Albuminuria (KDIGO A2)	**Wakasugi,S.2022**	cross-sectional analysis	OR	999	0.84 (0.76-0.93)	0.78 (0.58–1.05)
	**Chai,S.2022**	cross-sectional analysis	OR	1014	0.58 (0.52,0.65)	
	**Yoo,J.H.2020**	cross-sectional analysis	OR	866	0.97(0.95-1.04)	
Mild nonproliferative diabetic retinopathy(NPDR)	**Lu,J.2018**	cross-sectional analysis	OR	3262	0.93 (0.87–0.99)	
Carotid artery vasculopathy	**Lu,J.2020**	cross-sectional analysis	OR	2215	0.936 (0.878-0.998)	
lower extremity atherosclerotic disease	**Li,J.2020**	cross-sectional analysis	OR	336	0.81 (0.70-0.92)	0.86 (0.82-0.91)
	**Wang,Y.2022(2)**	cohort study	OR*	1351	0.87 (0.82–0.92)	
	**Li,F.2020#**	cross-sectional analysis	OR	740	0.86 (0.64–1.16)	
Diabetic peripheral neuropathy	**Mayeda,L.2020**	cross-sectional analysis	OR	105	0.81 (0.66-1.01)	0.77 (0.71–0.84)
	**Guo,Q.Y.2020**	cross-sectional analysis	OR	466	0.78 (0.71-0.87)	
	**Zhao,W.2022**	cross-sectional analysis	OR	206	0.67(0.54-0.84)	
Mild pain of painful diabetic neuropathy	**Yang,J.2021#**	cross-sectional analysis	OR	364	0.80 (0.50-1.28)	
Cardiovascular autonomic neuropathy	**Guo,Q.2020**	cross-sectional analysis	OR	349	0.74 (0.64-0.83)	0.81 (0.68-0.97)

	**Kim,M.Y.2021**	cross-sectional analysis	OR	284	0.89 (0.79–1.00)

Footnote: *effect value OR converted by HR; # 10% raise of TIR comparison converted by TIR quantiles.

S10. Adjusted covariates and adjusted model.

Author/Year	Age	Sex	Mean HbA1c	Baseline HbA1c	Duration of diabetes	BMI	TG/TC/HDL-C/LDL-C	Baseline systolic pressure/diastolic pressure	Baseline drug treatment for diabetes	Baseline use statins/aspirin	Adjusted model
Li,F.2020	*	*		*			*	*			logistic regression models
Guo,Q.Y.2020	*	*		*	*	*	*	*			logistic regression models
Yoo,J.H.2020	*	*		*	*	*	*	*			logistic regression models
Li,J.2020	*	*		*	*	*	*	*	*		logistic regression models
Lu,J.2020	*	*		*	*	*	*	*		*	logistic regression models
Lu,J.2021	*	*			*	*	*	*	*	*	cox proportional hazards model
Lu,J.2018	*	*		*	*	*	*	*			logistic regression models
Guo,J.2021	*	*		*		*				*	logistic regression models
Yang,J.2021	*	*		*		*	*		*		logistic regression

											models
Kim,M.Y.2021	*	*	*		*				*	*	logistic regression models
Guo,Q.2020	*	*		*	*	*	*	*			logistic regression models
Huang,R.2022	*	*		*	*	*	*				logistic regression models
Chai,S.2022	*	*	*		*	*	*	*			logistic regression models
Yin,X.2022	*	*	*		*	*					logistic regression models
Wang,Y.2022	*	*			*	*	*	*			logistic regression models
Wang,Y.2022 (2)	*	*			*	*	*	*		*	cox proportional hazards model
Shen,Y.2022	*	*		*			*		*	*	cox proportional hazards model
Xie,P.2022	*	*		*	*	*	*				logistic regression models
Zhao,W.2022	*	*		*	*	*	*	*			logistic regression models
Mayeda,L.2020	*	*			*	*			*		logistic regression models
Wakasugi,S.2021	*	*	*		*	*	*	*	*	*	logistic regression models
Richard,	*	*	*		*				*		cox proportional

B.2023										hazards model
C. Xu.2025	*	*	*	*	*	*	*	*		logistic regression models
Q. Zhang.2025	*	*	*	*	*	*	*	*	*	cox proportional hazards model

S11. NOS assessments

Author/Year	Representativeness of the exposed cohort	Selection of the non exposed cohort	Ascertainment of exposure	Demonstration that outcome of interest was not present at start of study	Comparability of cohorts on the basis of the design or analysis	Assessment of outcome	Was follow-up long enough for outcomes to occur	Adequacy of follow up of cohorts	Total scores
Li,F.2020	*	*	*	*	**	*		*	8
Guo, Q. Y. 2020	*	*	*	*	**	*		*	8
Yoo,J.H.2020	*	*	*	*	*	*		*	7
Li,J.2020	*	*	*	*	**	*		*	8
Lu,J.2020	*	*	*	*	**	*	*	*	9
Lu,J.2021	*	*	*	*	**	*	*	*	9
Lu,J.2018	*	*	*		**	*	*	*	8
Guo,J.2021	*	*	*	*	**	*	*	*	9
Yang,J.2021	*	*	*	*	*	*		*	7
Kim,M.Y.2021	*	*	*	*	**	*	*	*	9
Guo,Q.2020	*	*	*	*	**	*		*	8
Huang,R.2022	*	*	*	*	**	*		*	8
Chai,S.2022	*	*	*	*	**	*		*	8
Yin,X.2022	*	*	*	*	**	*		*	8

Wang,Y.2022	*	*	*	*	**	*		*	8
Wang,Y.2022 (2)	*	*	*	*	**	*	*	*	9
Shen,Y.2022	*	*	*	*	**	*	*	*	9
Xie,P.2022	*	*	*	*	**		*	*	8
Zhao,W.2022	*	*	*	*	**	*		*	8
Mayeda,L.2020	*	*	*	*	**	*		*	8
Wakasugi,S.2021	*	*	*	*	**	*		*	8
Richard, B.2023	*	*	*	*	*	*	*	*	8
C. Xu.2025	*	*	*	*	*	*	*	*	8
Q. Zhang.2025	*	*	*	*	*	*	*	*	8

S12. Meta-analyses of observational studies.

Outcome	Study ID	10% TIR increase OR (95% CI)	Participants	Event	Merged OR (95% CI)
All-cause mortality	**Xie,P.2022**	0.88 (0.78–0.98)	7802	1179	0.88 (0.82–0.93)
	**Lu,J.2021**	0.91 (0.88–0.94)*			
	**Q. Zhang.2025**	0.83 (0.78-0.89)*			
Amputation	**Xie,P.2022**	0.98 (0.96–0.99)	358	70	0.95 (0.88–1.03)
	**Yin,X.2022**	0.90 (0.81–0.98)			
Vision-threatening DR	**Lu,J.2018**	0.91 (0.85–0.98)	4261	266	0.93 (0.87–0.98)
	**Wakasugi,S.2021**	0.97 (0.86-1.09)			
Diabetic retinopathy	**Lu,J.2018**	0.92 (0.88–0.96)	5590	1476	0.92 (0.89–0.95)
	**Wang,Y.2022**	0.93 (0.88-0.97)			
	**C. Xu.2025**	0.53 (0.29,0.94)			
Albuminuria (KDIGO A3)	**Wakasugi,S.2022**	0.81 (0.72-0.90)	3287	603	0.58 (0.27–1.21)
	**Chai,S.2022**	0.26 (0.18,0.38)			
	**Q. Zhang.2025**	0.86 (0.81-0.92)			
Albuminuria (KDIGO A2)	**Wakasugi,S.2022**	0.84 (0.76-0.93)	2879	1160	0.78 (0.58–1.05)
	**Chai,S.2022**	0.58 (0.52,0.65)			
	**Yoo,J.H.2020**	0.97(0.95-1.04)			
lower extremity atherosclerotic disease	**Li,J.2020**	0.81 (0.70-0.92)	1687	511	0.86 (0.82–0.91)
	**Wang,Y.2022 (2)**	0.87 (0.82–0.92)*			
Diabetic peripheral neuropathy	**Li,F.2020[#]**	0.86 (0.64–1.16)	1517	303	0.77 (0.71–0.84)

Cardiovascular autonomic neuropathy	**Mayeda,L.2020**	0.81 (0.66-1.01)	633	205	0.81 (0.68–0.97)
	**Guo,Q.Y.2020**	0.78 (0.71-0.87)			
	**Zhao,W.2022**	0.67(0.54-0.84)			
	**Guo,Q.2020**	0.74 (0.64-0.83)			
	**Kim,M.Y.2021**	0.89 (0.79–1.00)			

Footnote: *effect value OR converted by HR; # 10% raise of TIR comparison converted by TIR quantile.

S13. Sensitivity analyses

S13.1 Leave-one-out analysis

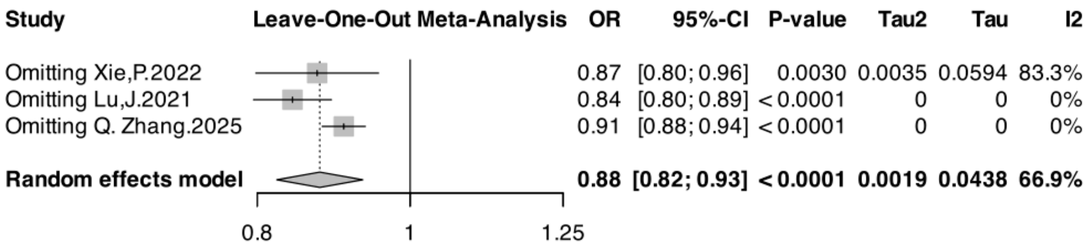

FigureS1.TIR, time in range, OR, odds ratio,OR date refer to the association of each 10% raise of TIR with risk of incident all-cause mortality.

(2) Amputation

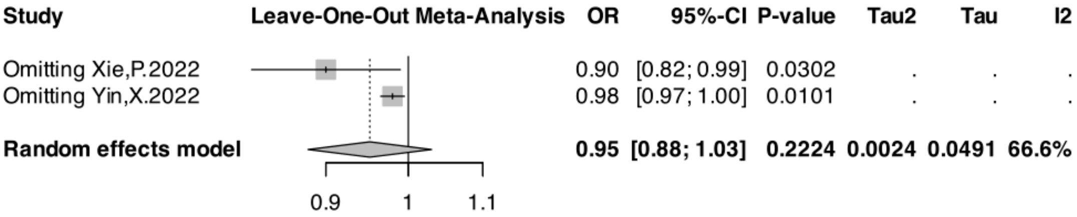

FigureS2.TIR, time in range, OR, odds ratio,OR date refer to the association of each 10% raise of TIR with risk of incident amputation.

(3) Vision-threatening DR

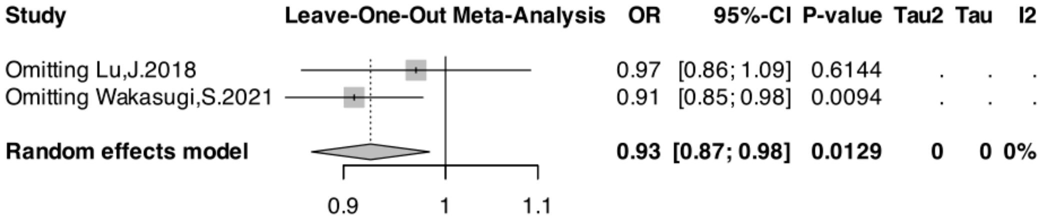

FigureS3.TIR, time in range, OR, odds ratio,OR date refer to the association of each 10% raise of TIR with risk of vision-threatening DR.

(4) Diabetic retinopathy

Study	OR	95%-CI	P-value	Tau2	Tau	I2
Omitting Lu,J.2018	0.76	[0.45; 1.29]	0.3092	0.1128	0.3358	71.3%
Omitting Wang,Y.2022	0.76	[0.45; 1.27]	0.2915	0.1068	0.3268	70.2%
Omitting C. Xu.2025	0.92	[0.89; 0.95]	< 0.0001	0	0	0%
**Random effects model**	**0.92**	**[0.89; 0.95]**	**< 0.0001**	**< 0.0001**	**0.0016**	**43.4%**

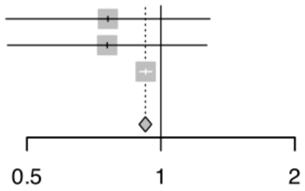

FigureS4.TIR, time in range, OR, odds ratio,OR date refer to the association of each 10% raise of TIR with risk of diabetic retinopathy.

(5) Albuminuria (KDIGO A3)

Study	OR	95%-CI	P-value	Tau2	Tau	I2
Omitting Wakasugi,S.2022	0.48	[0.15; 1.55]	0.2195	0.6968	0.8348	97.4%
Omitting Chai,S.2022	0.85	[0.80; 0.90]	< 0.0001	0	0	0%
Omitting Q. Zhang.2025	0.47	[0.15; 1.42]	0.1784	0.6259	0.7911	96.9%
**Random effects model**	**0.58**	**[0.27; 1.21]**	**0.1469**	**0.4176**	**0.6462**	**94.8%**

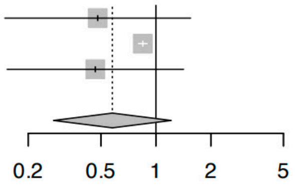

FigureS5.TIR, time in range, OR, odds ratio,OR date refer to the association of each 10% raise of TIR with risk of Albuminuria (KDIGO A3).

(6) Albuminuria (KDIGO A2)

Study	OR	95%-CI	P-value	Tau2	Tau	I2
Omitting Wakasugi,S.2022	0.75	[0.45; 1.24]	0.2677	0.1303	0.3610	98.6%
Omitting Chai,S.2022	0.91	[0.79; 1.05]	0.1842	0.0088	0.0936	84.6%
Omitting Yoo,J.H.2020	0.70	[0.49; 1.00]	0.0527	0.0656	0.2562	95.7%
**Random effects model**	**0.78**	**[0.58; 1.05]**	**0.1064**	**0.0680**	**0.2607**	**97.2%**

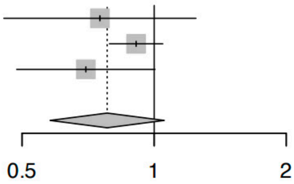

FigureS6.TIR, time in range, OR, odds ratio,OR date refer to the association of each 10% raise of TIR with risk of Albuminuria (KDIGO A2).

(7) Lower extremity atherosclerotic disease

Study	OR	95%-CI	P-value	Tau2	Tau	I2
Omitting Li,J.2020	0.87	[0.82; 0.92]	< 0.0001	.	.	.
Omitting Wang,Y.2022(2)	0.81	[0.71; 0.93]	0.0025	.	.	.
**Random effects model**	**0.86**	**[0.82; 0.91]**	**< 0.0001**	**0**	**0**	**0%**

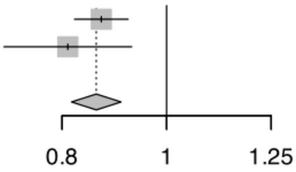

FigureS7.TIR, time in range, OR, odds ratio,OR date refer to the association of each 10% raise of TIR with risk of lower extremity atherosclerotic disease.

(8) Diabetic peripheral neuropathy

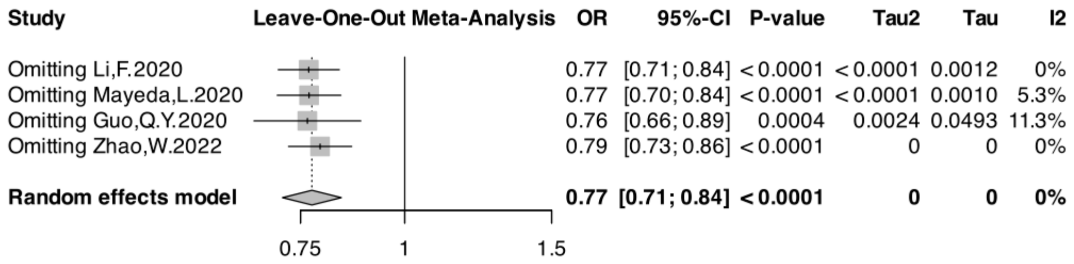

FigureS8.TIR, time in range, OR, odds ratio,OR date refer to the association of each 10% raise of TIR with risk of diabetic peripheral neuropathy.

(9) Cardiovascular autonomic neuropathy

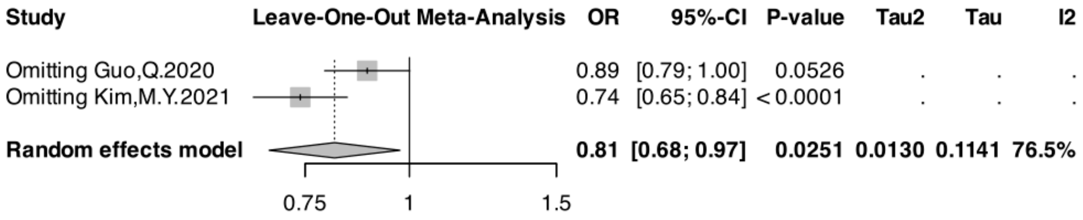

FigureS9.TIR, time in range, OR, odds ratio,OR date refer to the association of each 10% raise of TIR with risk of cardiovascular autonomic neuropathy.

S13.2 fixed effect model analysis

Outcome	Random effect model OR (95% CI)	Fixed effect model OR (95% CI)
All-cause mortality	0.88 (0.82-0.93)	0.89 (0.87-0.92)
Amputation	0.95 (0.88-1.03)	0.98 (0.96-0.99)
Vision-threatening diabetic retinopathy	0.93 (0.87-0.98)	0.93 (0.87-0.98)
Diabetic retinopathy	0.92 (0.89-0.95)	0.92 (0.89-0.95)
Albuminuria (KDIGO A3)	0.58 (0.27-1.21)	0.83 (0.78-0.87)
Lower extremity atherosclerotic disease	0.86 (0.82-0.91)	0.86(0.82-0.91)
Albuminuria (KDIGO A2)	0.78 (0.58-1.05)	0.89 (0.86-0.93)
Cardiovascular autonomic neuropathy	0.81 (0.68-0.97)	0.82 (0.75-0.89)
Diabetic peripheral neuropathy	0.77 (0.71-0.84)	0.77 (0.71-0.84)

S13.3 Sensitivity analysis excluding FCGM-based studies

Outcome	Sensitivity analysis (CGM-only)	Primary analysis (all studies)
All-cause mortality	0.87 (0.80–0.96), 2 studies	0.88 (0.82–0.93), 3 studies
Albuminuria (KDIGO A3)	0.85 (0.80–0.90), 2 studies	0.58 (0.27–1.21), 3 studies
Albuminuria (KDIGO A2)	0.91 (0.79–1.05), 2 studies	0.78 (0.58–1.05), 3 studies
Amputation	0.90 (0.81–0.98), 1 study (descriptive)	0.95 (0.88–1.03), 2 studies
MACE	Not assessable (0 studies)	0.92 (0.88–0.96), 1 study (descriptive)

S14. Grade assessment criteria for Meta-analyses of observational studies.

Degradation factor	Degradation criteria	Operating rules
Publication bias	Authors of systematic reviews should suspect publication bias when studies are uniformly small, particularly when sponsored by the industry	-1
Imprecision	1.CI overlaps no effect<br>2. wide CIs	1 or 2，-1<br>1 and 2，-2
Risk of bias	Based on the NOS bias risk assessment results:<br>1. All or most (2/3) of the studies comes from low risk<br>2. Most (2/3) of the studies comes from medium risk<br>3. Most (2/3) of the studies comes from high-risk sources	The first point, no penalty<br>The second point, -1<br>The third point, -2
Inconsistency	1. Point estimates vary widely across studies<br>2. Confidence intervals (CIs) show minimal or no overlap<br>3. The statistical test for heterogeneity which tests the null hypothesis that all studies in a meta-analysis have the same underlying magnitude of effect shows a low P-value<br>4. The I^2 which quantifies the proportion of the variation in point estimates due to among-study differences is large	$\leq$ 2 points is satisfied，-1<br>> 2 points is satisfied，-2
Indirectness	1. Population differences<br>2. Differences in intervention measures<br>3. Differences in outcome measurement<br>4. Indirect comparison	One of the four points is satisfied,-1<br>Two of the four points is satisfied,-2
**Upgradation factor**	**Upgrade criteria**	**Operating rules**
Large effect size	Significant or very significant effect values of included studies	OR ≤0.9, +1<br>OR ≤0.5, +2

Dose response gradient	Linearity of the dose-response relationship between TIR and adverse outcomes	+1
Plausible confounding can increase confidence in estimated effects	Some observational studies may fail to consider all confounding factors in their adjusted analysis.	+1

S15. Random effect model analysis forest plots

FigureS1:The association between TIR and all-cause mortality based on each 10% raise of TIR contrast.

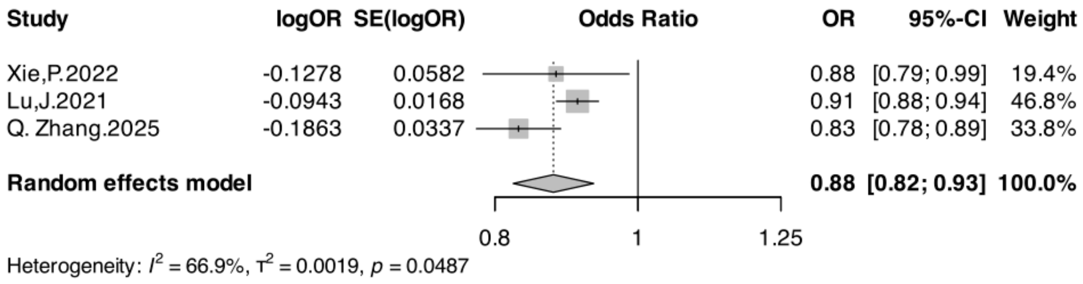

FigureS1.TIR, time in range, OR, odd ratio,OR date refer to the association of each 10% raise of TIR with risk of incident all-cause mortality.

FigureS2:The association between TIR and amputation besed on each 10% raise of TIR contrast.

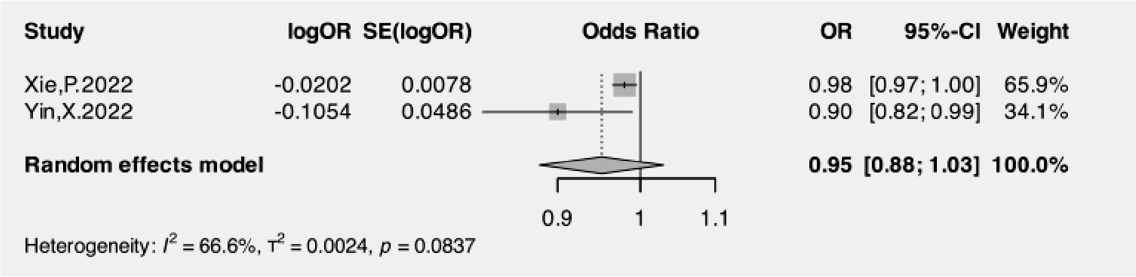

FigureS2.TIR, time in range, OR, odd ratio,OR date refer to the association of each 10% raise of TIR with risk of incident amputation.

FigureS3:The association between TIR and vision-threatening DR besed on each 10% raise of TIR contrast.

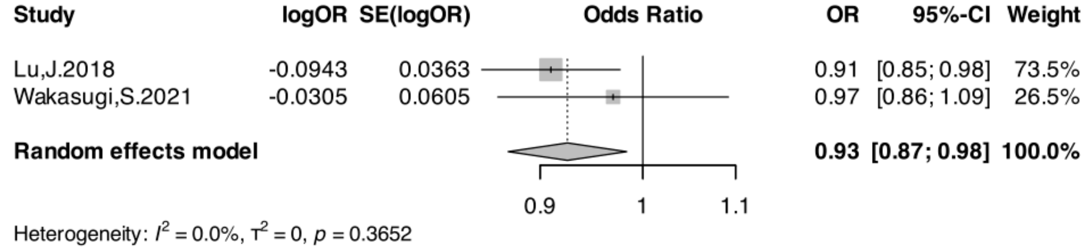

FigureS3.TIR, time in range, OR, odd ratio,OR date refer to the association of each 10% raise of TIR with risk of vision-threatening DR.

FigureS4:The association between TIR and diabetic retinopathy besed on each 10% raise of TIR contrast.

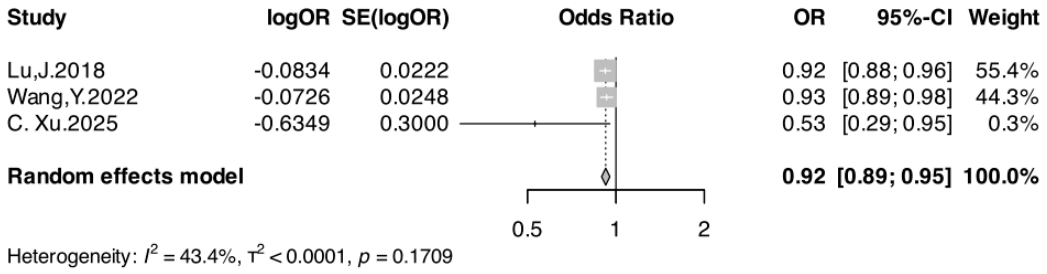

FigureS4.TIR, time in range, OR, odd ratio,OR date refer to the association of each 10% raise of TIR with risk of diabetic retinopathy.

FigureS5:The association between TIR and Albuminuria (KDIGO A3) besed on each 10% raise of TIR contrast.

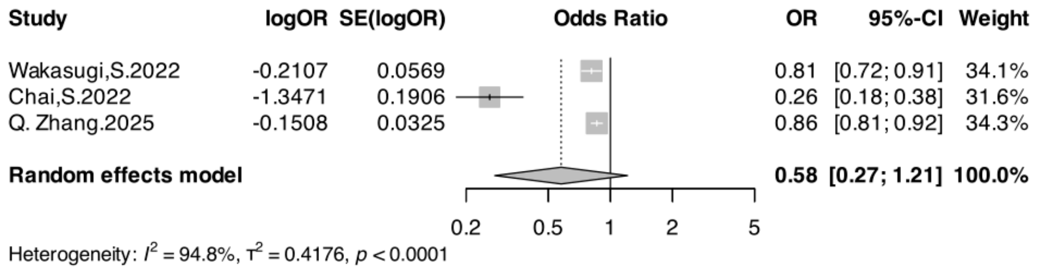

FigureS5.TIR, time in range, OR, odd ratio,OR date refer to the association of each 10% raise of TIR with risk of Albuminuria (KDIGO A3).

FigureS6:The association between TIR and Albuminuria (KDIGO A2) besed on each 10% raise of TIR contrast.

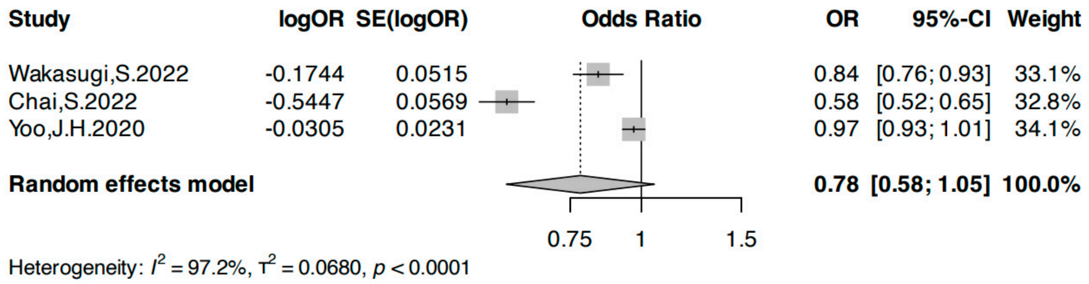

FigureS6.TIR, time in range, OR, odd ratio,OR date refer to the association of each 10% raise of TIR with risk of Albuminuria (KDIGO A2).

FigureS7:The association between TIR and lower extremity atherosclerotic disease besed on each 10% raise of TIR contrast.

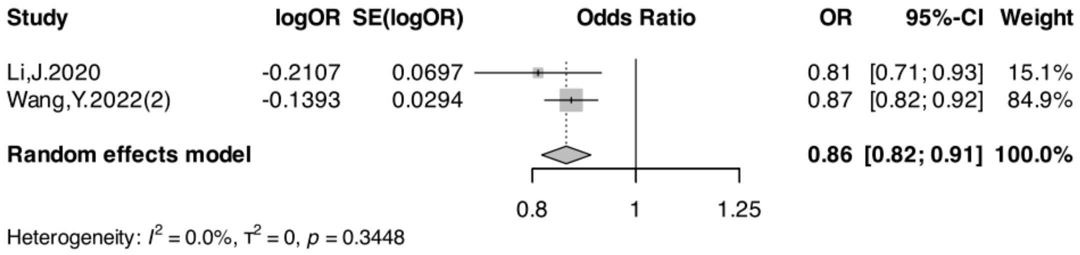

FigureS7.TIR, time in range, OR, odd ratio,OR date refer to the association of each 10% raise of TIR with risk of lower extremity atherosclerotic disease.

FigureS8:The association between TIR and diabetic peripheral neuropathy besed on each 10% raise of TIR contrast.

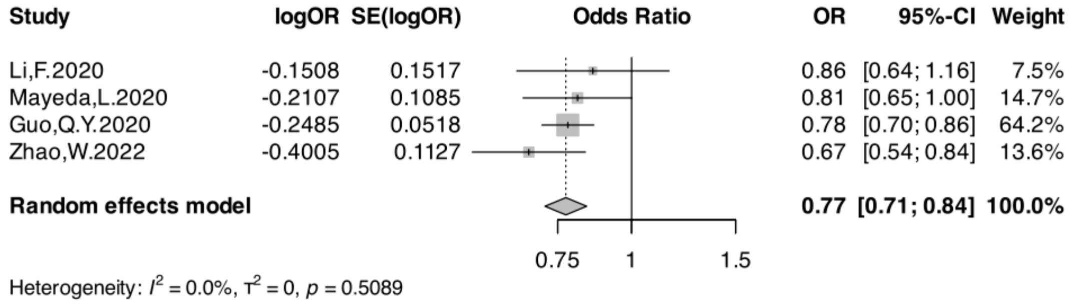

FigureS8.TIR, time in range, OR, odd ratio,OR date refer to the association of each 10% raise of TIR with risk of diabetic peripheral neuropathy.

FigureS9:The association between TIR and cardiovascular autonomic neuropathy besed on each 10% raise of TIR contrast.

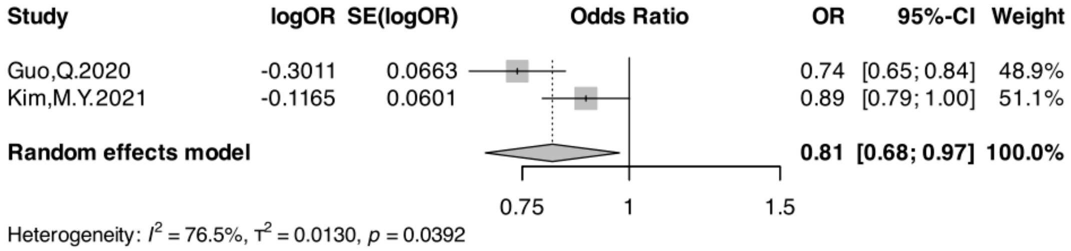

FigureS9.TIR, time in range, OR, odd ratio,OR date refer to the association of each 10% raise of TIR with risk of cardiovascular autonomic neuropathy.

S16. Fixed effect model analysis forest plots

FigureS1:The association between TIR and all-cause mortality based on each 10% raise of TIR contrast.

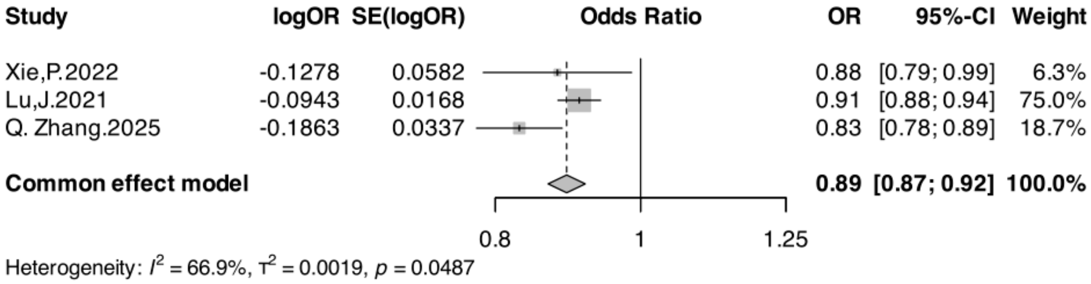

FigureS1.TIR, time in range, OR, odd ratio,OR date refer to the association of each 10% raise of TIR with risk of incident all-cause mortality.

FigureS2:The association between TIR and amputation based on each 10% raise of TIR contrast.

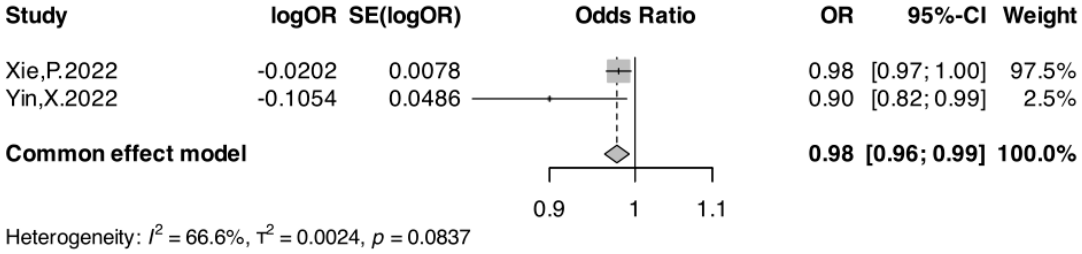

FigureS2.TIR, time in range, OR, odd ratio,OR date refer to the association of each 10% raise of TIR with risk of incident amputation.

FigureS3:The association between TIR and vision-threatening DR based on each 10% raise of TIR contrast.

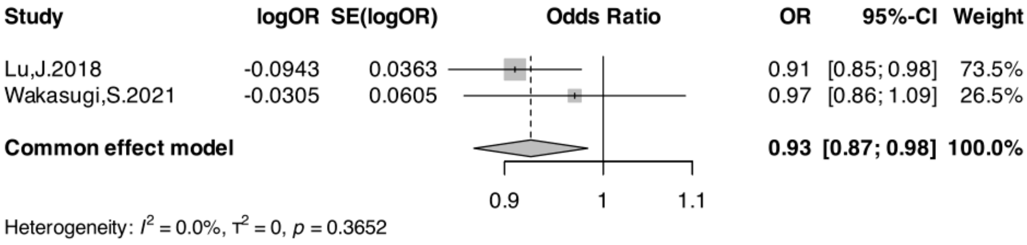

FigureS3.TIR, time in range, OR, odd ratio,OR date refer to the association of each 10% raise of TIR with risk of vision-threatening DR.

FigureS4:The association between TIR and diabetic retinopathy based on each 10% raise of TIR contrast.

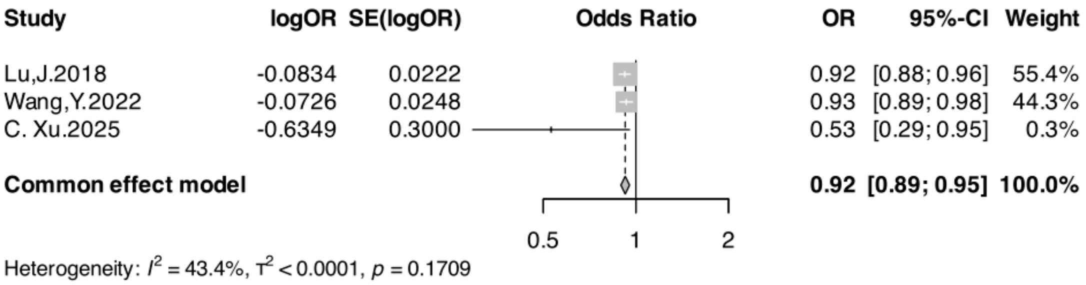

FigureS4.TIR, time in range, OR, odd ratio,OR date refer to the association of each 10% raise of TIR with risk of diabetic retinopathy.

FigureS5:The association between TIR and Albuminuria (KDIGO A3) based on each 10% raise of TIR contrast.

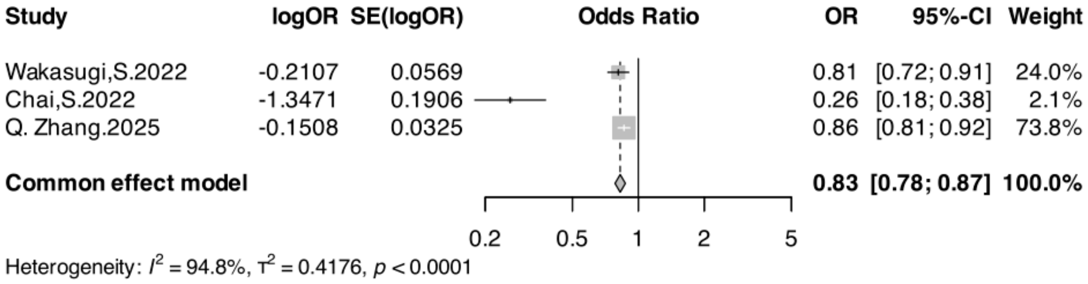

FigureS5.TIR, time in range, OR, odd ratio,OR date refer to the association of each 10% raise of TIR with risk of Albuminuria (KDIGO A3).

FigureS6:The association between TIR and Albuminuria (KDIGO A2) based on each 10% raise of TIR contrast.

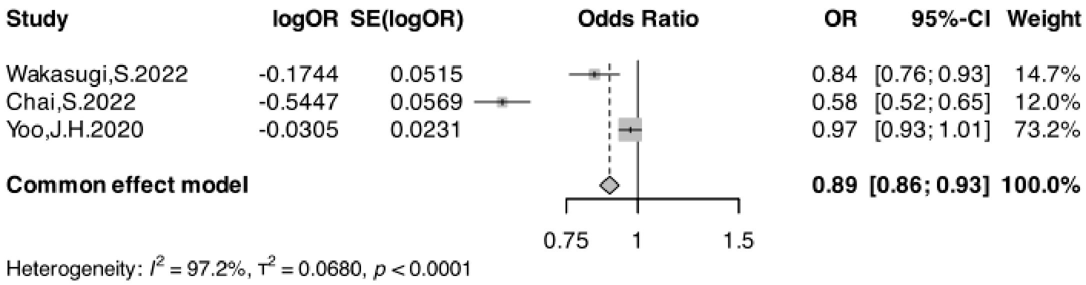

FigureS6.TIR, time in range, OR, odd ratio,OR date refer to the association of each 10% raise of TIR with risk of Albuminuria (KDIGO A2).

FigureS7:The association between TIR and lower extremity atherosclerotic disease based on each 10% raise of TIR contrast.

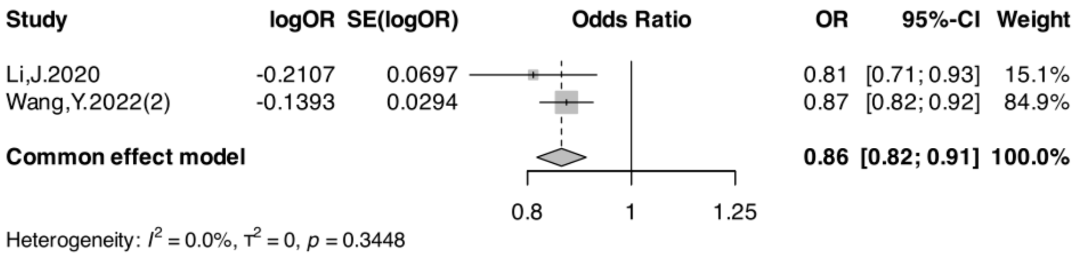

FigureS7.TIR, time in range, OR, odd ratio,OR date refer to the association of each 10% raise of TIR with risk of lower extremity atherosclerotic disease.

FigureS8:The association between TIR and diabetic peripheral neuropathy based on each 10% raise of TIR contrast.

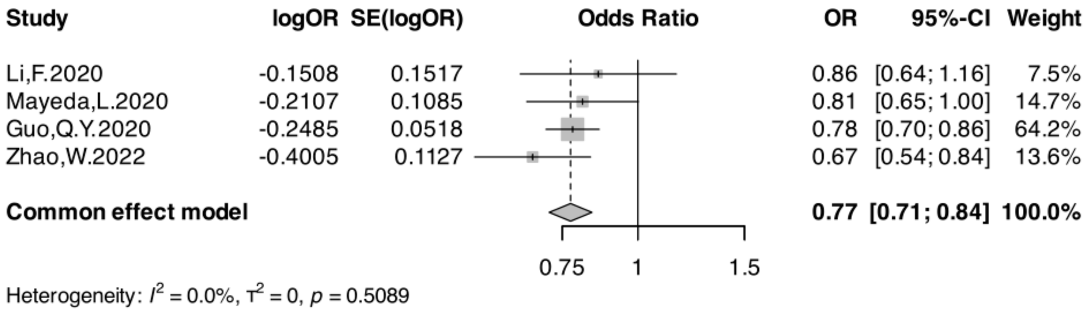

FigureS8.TIR, time in range, OR, odd ratio,OR date refer to the association of each 10% raise of TIR with risk of diabetic peripheral neuropathy.

FigureS9:The association between TIR and cardiovascular autonomic neuropathy based on each 10% raise of TIR contrast.

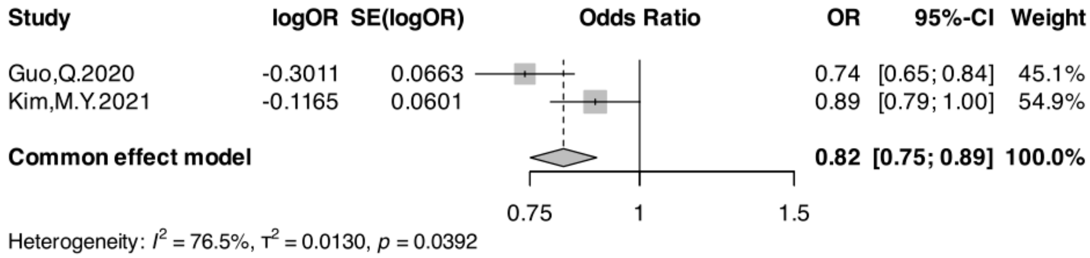

FigureS9.TIR, time in range, OR, odd ratio,OR date refer to the association of each 10% raise of TIR with risk of cardiovascular autonomic neuropathy.
